# Pterostilbene exerts anticancer activity on non-small-cell lung cancer via activating endoplasmic reticulum stress

**DOI:** 10.1038/s41598-017-08547-0

**Published:** 2017-08-14

**Authors:** Zhiqiang Ma, Yang Yang, Shouyin Di, Xiao Feng, Dong Liu, Shuai Jiang, Wei Hu, Zhigang Qin, Yue Li, Jianjun Lv, Chongxi Fan, Xiaolong Yan, Xiaofei Li

**Affiliations:** 1 Department of Thoracic Surgery, Tangdu Hospital, The Fourth Military Medical University, 1 Xinsi Road, Xi’an, 710038 China; 2 Department of Endocrinology, The First Affiliated Hospital of Xi’an Jiaotong University, 277 Yanta West Road, Xi’an, 710061 China; 3 Key Laboratory of Resource Biology and Biotechnology in Western China, Ministry of Education, Faculty of Life Sciences, Northwest University, 229 Taibai North Road, Xi’an, 710069 China; 4 Department of Biomedical Engineering, The Fourth Military Medical University, 169 Changle West Road, Xi’an, 710032 China; 5 Department of Cardiovascular Surgery, Xijing Hospital, The Fourth Military Medical University, 127 Changle West Road, Xi’an, 710032 China; 6 State Key Laboratory of Cardiovascular Disease, Fuwai Hospital, National Center for Cardiovascular Diseases, Chinese Academy of Medical Sciences, Peking Union Medical College, 167 Beilishi Road, Beijing, 100037 China; 7 Department of Aerospace Medicine, The Fourth Military Medical University, 169 Changle West Road, Xi’an, 710032 China

## Abstract

Pterostilbene (PT), the natural dimethylated analog of resveratrol (RSV), is a potent anticarcinogen for non-small-cell lung cancer (NSCLC), but its anti-NSCLC mechanisms remain unclear. In this study, we show that PT treatment time- and dose-dependently enhanced the endoplasmic reticulum stress (ERS) signaling (i.e., p-PERK, IRE1, ATF4, CHOP), thus decreasing the cell viability and inducing apoptosis in human PC9 and A549 NSCLC cell lines. Moreover, the decreased migratory and adhesive abilities, downregulation of intracellular glutathione (GSH) level, enhanced reactive oxygen species (ROS) generation, Caspase 3 activity and mitochondrial membrane depolarization were observed in NSCLC cells treated with PT. These effects were reversed by CHOP siRNA which inhibited the ERS signaling pathway, but were promoted by thapsigargin (a classical ERS inducer) *in vitro*. Besides, *in vivo* studies also verify that PT exerted anticancer activity by mobilizing ERS signaling and apoptosis-related proteins, and these effects were enhanced by thapsigargin. Therefore, ERS activation may represent a new mechanism of anti-NSCLC action by PT, and a novel therapeutic intervention for lung cancer.

## Introduction

Lung cancer, representing 19% of all cancer deaths worldwide^1^, is the most frequently diagnosed cancer and the largest number of malignancy deaths among males and females^2, 3^. More than 85% of lung cancer cases are classified as non-small-cell lung cancer (NSCLC)^4^. Though great strides have been made in NSCLC therapy, the predicted 5-year survival rate is only 15.9%^5^. The high death toll from NSCLC and unsatisfactory outcomes of treatments thus have spurred us to understand their molecular basis and find novel agents especially from natural materials with few harmful effects in NSCLC therapy^6, 7^. Pterostilbene (PT), a potential anticarcinogen lack of toxic and undesirable side effects, has attracted more attention^8^.

Stilbenes (e.g., RSV and PT) are a group of naturally phenolic agents with diverse pharmacological actions, especially the anticancer activity^9, 10^. PT is abundant in a variety of berries, even some blueberries contain up to 15 μg PT per cup^11^. As the emerging tumor suppressor, studies have reported that PT is superior to RSV^9, 10^, and exerts potent anticancer activities against various malignancies, including breast^12^, ovarian^13^, esophageal^10^, oral^14^, prostate^15^, pancreatic^16^, liver^17^, colon^9^, and blood cancers^18, 19^, etc. Moreover, PT is also a potent anticancer compound against NSCLC^8, 20, 21^, but its anti-NSCLC mechanisms have not yet been clearly elucidated.

The endoplasmic reticulum (ER) is perinuclear, cytosolic compartment for the Ca^2+^ storage, lipids or proteins synthesis, and folding and modification of proteins^22^. Numerous insulted conditions, such as nutrient deprivation, ER Ca^2+^ depletion, hypoxia or oxidative stress, infections, and drug treatments, may perturb ER, induce unfolded proteins accumulation, then lead to ER stress (ERS)^22, 23^. Cancers are often challenged by hypoxia and lack of nutrients during progression, thus resulting in ERS^23^. To survive those hostile environments, unfolded protein response (UPR) is activated to restore ER proteostasis via three main UPR sensor proteins, including (i) the protein kinase RNA-like ER kinase (PERK), (ii) inositol-requiring kinase 1 (IRE1), and (iii) activating transcription factor 6 (ATF6), which are all controlled by the ER chaperone glucose regulated protein 78 (GRP78/Bip)^22–24^. However, persistent ERS could tip the balance towards apoptosis and leads to cell death^22^. ATF4 stimulated by phospho-PERK (p-PERK) activates the death effector, transcription of C/EBP homologous protein (CHOP/GADD153), thus promoting mitochondria-initiated apoptosis regulated by Bcl2 family proteins, evidenced by repression of anti-apoptotic factors (e.g., Bcl2, Bcl-xL, Mcl-1) and initiation of pro-apoptotic factors (e.g., Bax, Bak, Bim, PUMA)^2, 7, 10, 22^. Therefore, inducers of ERS (e.g., thapsigargin and tunicamycin) may provide efficient cancer therapies in cancer cells, and bortezomib^2, 7^, the first proteasome inhibitor for cancer therapy to be approved by the US Food and Drug Administration, functions as an inducer of ERS^23, 25, 26^. Experimentally, PT was proven as a potent ERS activator against esophageal cancer via inducing apoptosis-related cell death^10^, but the effects of ERS signaling in the anti-NSCLC actions of PT have not been examined. In this study, we assessed the anticancer activity of PT in NSCLC and explored the role of ERS signaling in PT treatment.

## Results

### Inhibition of cell viability and induction of apoptosis by PT treatment on PC9 and A549 cells

**Figure 1:**
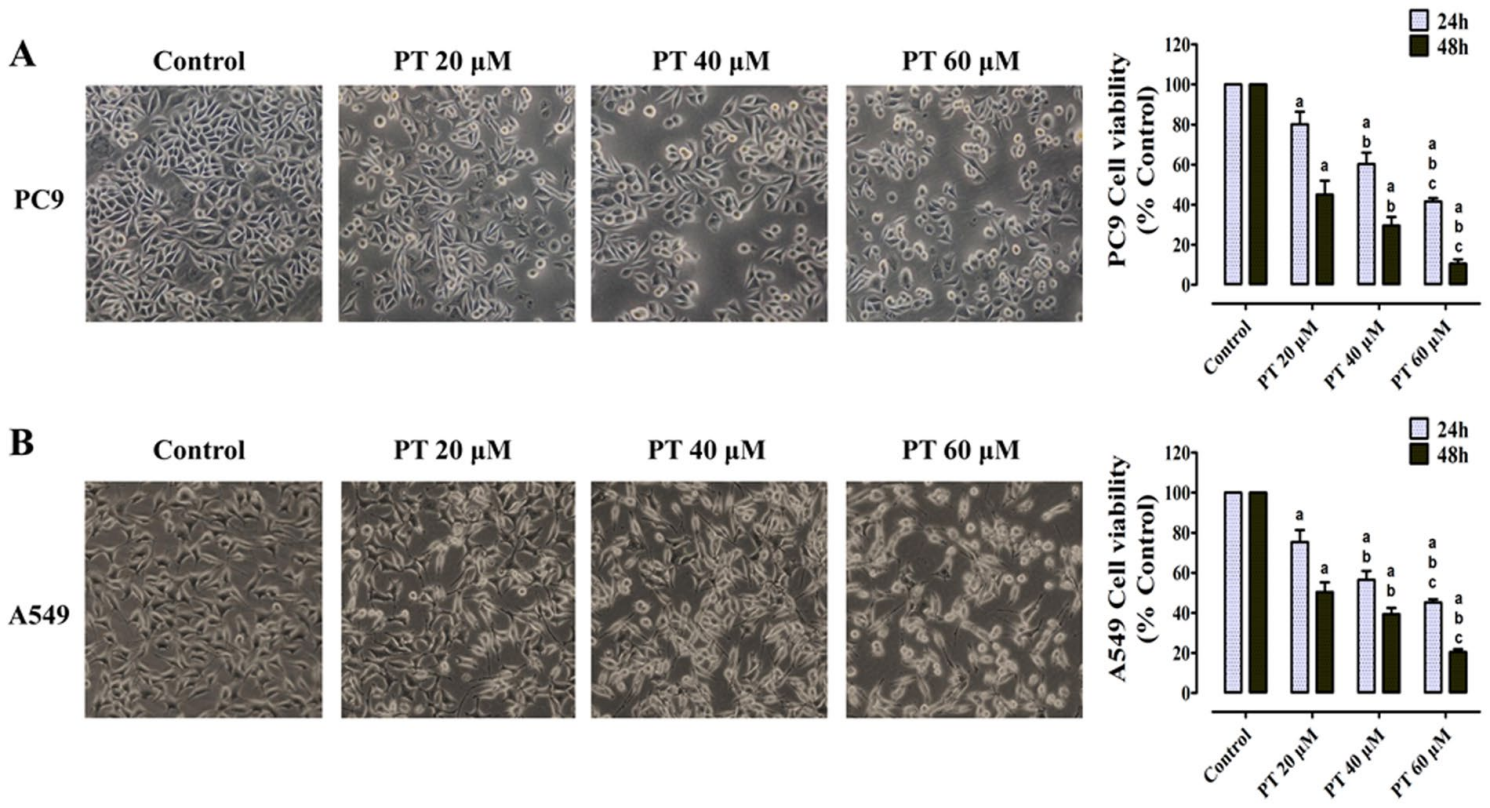
Effect of PT treatment on the viability and morphology of PC9 and A549 cells. Both cell lines were treated with increasing concentrations of PT (20, 40, and 60 μM) and assessed at time points of 24 and 48 h. The cell viability was analyzed by CCK-8 and expressed as OD values (% Control). The morphology of both cells were observed under an inverted phase-contrast microscope after the cells were treated for 24 h, and images were obtained. Significant cell shrinkage and a decreased cellular attachment rate were observed in the PT treatment groups with a dose dependent manner. All of the results were expressed as the mean ± SD; n = 6. ^a^P < 0.05 *vs*. the control group, ^b^P < 0.05 *vs*. the 20 μM PT-treated group, ^c^P < 0.05 *vs*. the 40 μM PT-treated group.

**Figure 2:**
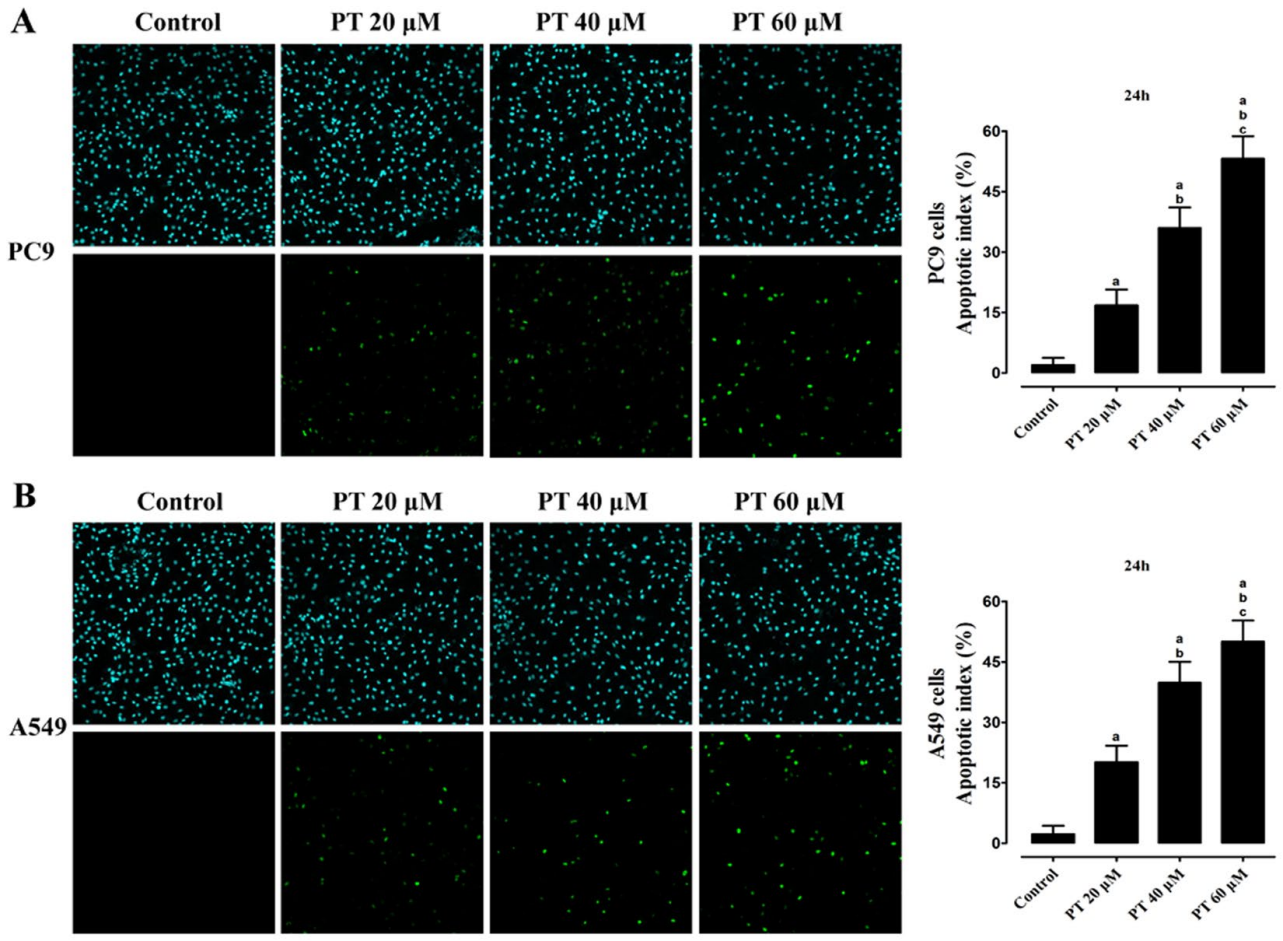
Effect of PT treatment on the apoptosis of PC9 and A549 cells (24 h). After treatment on both cell lines (**A** for PC9, **B** for A549), the apoptotic index of PT-treated groups exerted a dose-dependent increase in induction of apoptosis. The upper panel showed the cell nucleus (*blue*) and the lower panel displayed the apoptotic cells (*green*). All of the results were expressed as the mean ± SD; n = 6. ^a^P < 0.05 *vs*. the control group, ^b^P < 0.05 *vs*. the 20 μM PT-treated group, ^c^P < 0.05 *vs*. the 40 μM PT-treated group.

To investigate whether PT has the anticancer effect on NSCLC, the CCK-8 assay was employed to evaluate its cytotoxic role on PC9 and A549 cells (Fig. 1). Treatment on cells for 24 h or 48 h with 20 μM, 40 μM, and 60 μM PT inhibited cell viability in a dose- and time- dependent manner, and the IC50 values of PT at 24 h and 48 h were approximately 50.09 μM and 27.35 μM in PC9 cells; and 52.01 μM and 24.12 μM in A549 cells, respectively. Microscopic images (PC9 in Fig. 1A and A549 in Fig. 1B) indicated that PT treatment resulted in significant cell shrinkage and decreased cellular attachment rate compared with their control groups. After treatment with 20, 40, and 60 μM PT for 24 h, the apoptotic index dose-dependently increased to 16.75 ± 3.98%, 35.96 ± 5.81%, and 53.18 ± 6.53% in PC9 cells; and 20.16 ± 4.05%, 39.84 ± 6.21%, and 50.07 ± 7.19% in A549 cells, respectively. This effect was both estimated by TUNEL assay and Annexin V-FITC/PI assay (Fig. 2 and Supplementary Figure 2A). Moreover, to investigate whether PT has inhibitory role on HBE cells (normal human bronchial epithelial cells), we found that PT at low concentrations (20 and 40 μM) had weaker inhibitory effect on HBE cells than on NSCLC cells, but PT at higher concentration (60 μM) had similar inhibitory effect on both HBE and NSCLC cells (Supplementary Figure 1A).

### PT treatment promotes mitochondrial membrane depolarization, Caspase 3 activity, ROS generation and reduces GSH level on PC9 and A549 cells

**Figure 3:**
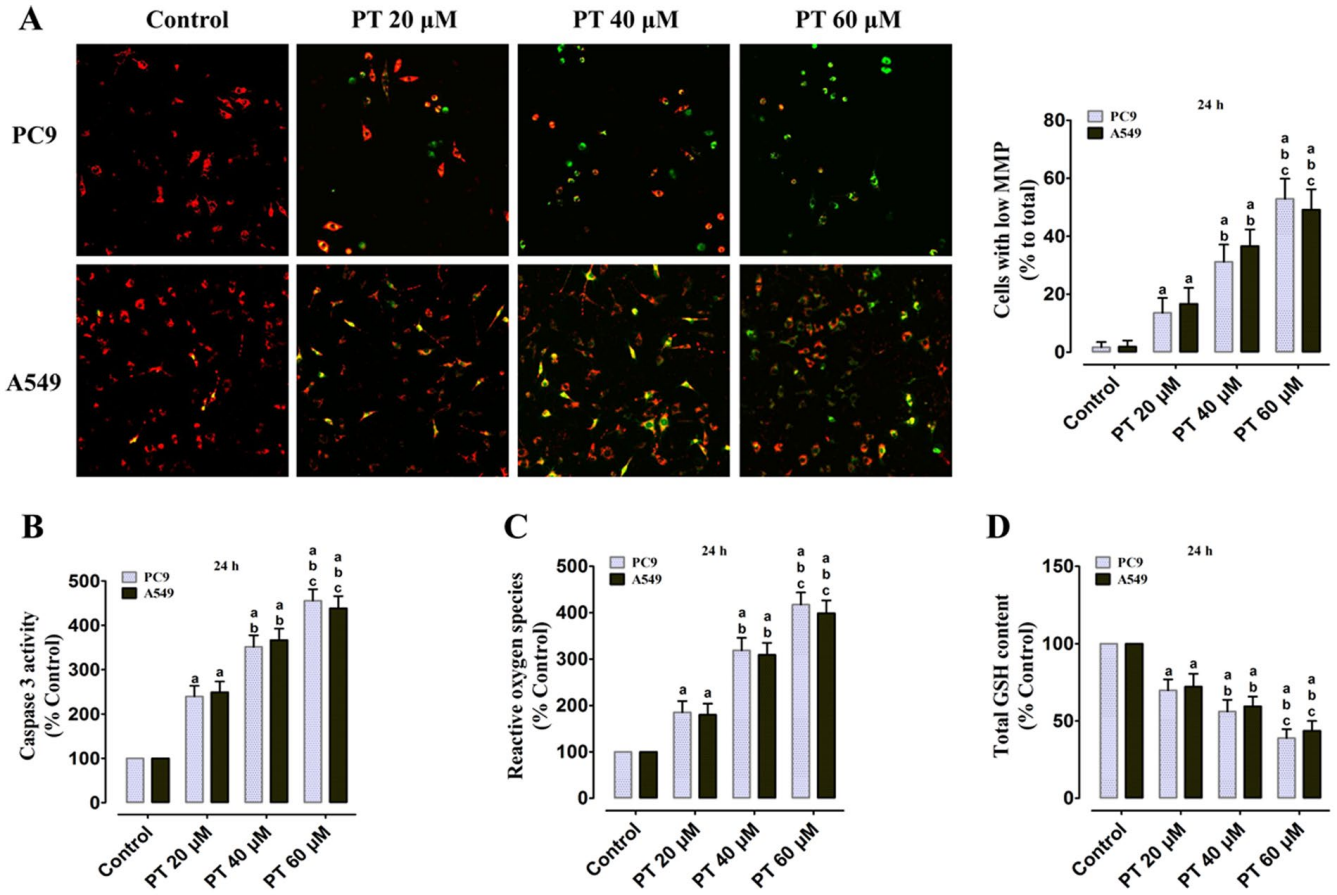
Effect of PT treatment on the depolarization of mitochondrial membrane, Caspase 3 activity, ROS generation, and GSH level in PC9 and A549 cells (24 h). (**A**) Representative merged images of JC-1 red (*normal MMP*)/green (*low MMP*) cells, and the MMP levels were expressed as the proportion of cells with a low MMP. (**B**) Intracellular Caspase 3 activity; (**C**) ROS concentration; (**D**) Intracellular GSH level; and those three indexes in the control group were defined as 100%. All of the results were expressed as the mean ± SD; n = 6. ^a^P < 0.05 *vs*. the control group, ^b^P < 0.05 *vs*. the 20 μM PT-treated group, ^c^P < 0.05 *vs*. the 40 μM PT-treated group.

The changes in MMP was assessed by using JC-1 staining. After treatment with PT (20, 40, and 60 μM) for 24 h, the ratio of cells with low MMP (green flourescence), which is a sign of early stages of cell apoptosis, significantly increases in a dose-dependent manner compared with the control group (P < 0.05, Fig. 3A). These results suggest that PT treatment promotes mitochondrial membrane depolarization which are consistent with the outcomes of TUNEL assay and Annexin-V/PI assay. Furthermore, the Caspase 3 activity (Fig. 3B) significantly increased to 239.67 ± 22.59%, 351.33 ± 26.06%, and 455.61 ± 25.83% in PC9 cells; and 248.96 ± 24.31%, 366.67 ± 25.67%, and 438.67 ± 27.17% in A549 cells, respectively (P < 0.05, compared with the control group). z-DEVD-fmk, a Caspase 3 specific inhibitor, was applied to confirm the implication of Caspase 3 in the mechanism of PT-induced apoptosis on NSCLC cells. Pre-incubation with z-DEVD-fmk 30 μM for 1 h completely inhibited PT-induced Caspase 3 activity upregulation and abolished PT-induced cell death in NSCLC cells, suggesting that the PT-induced cell apoptosis is Caspase 3-dependent (P < 0.05, Supplementary Figure 2B); however, z-DEVD-fmk 30 μM alone did not affect cell viability and Caspase 3 activity compared with the control group (P > 0.05). Intracellular ROS production was analyzed based on the ROS-mediated conversion of non-fluorescent 2’,7’-DCFH-DA into fluorescent DCFH, which exhibits enhanced fluorescence intensity following the generation of intracellular reactive metabolites. PT treatments dose-dependently increase the generation of ROS to 185.33 ± 24.12%, 318.82 ± 27.11%, and 417.40 ± 26.34% in PC9 cells; and 180.10 ± 23.87%, 309.16 ± 25.85%, and 398.91 ± 27.67% in A549 cells, respectively (P < 0.05, compared with the control group, Fig. 3C). GSH is the major non-protein thiol in cells and maintains the cellular redox status. After treatment with PT (20, 40, and 60 μM) for 24 h, the intracellular GSH levels decreased to 69.83 ± 7.11%, 56.08 ± 7.56%, and 38.89 ± 5.84% in PC9 cells; and 72.18 ± 8.31%, 59.51 ± 6.20%, and 43.67 ± 6.33% in A549 cells in a dose-dependent manner (P < 0.05, compared with the control group, Fig. 3D).

### Inhibition of cell migration and adhesion by PT treatment on PC9 and A549 cells

**Figure 4:**
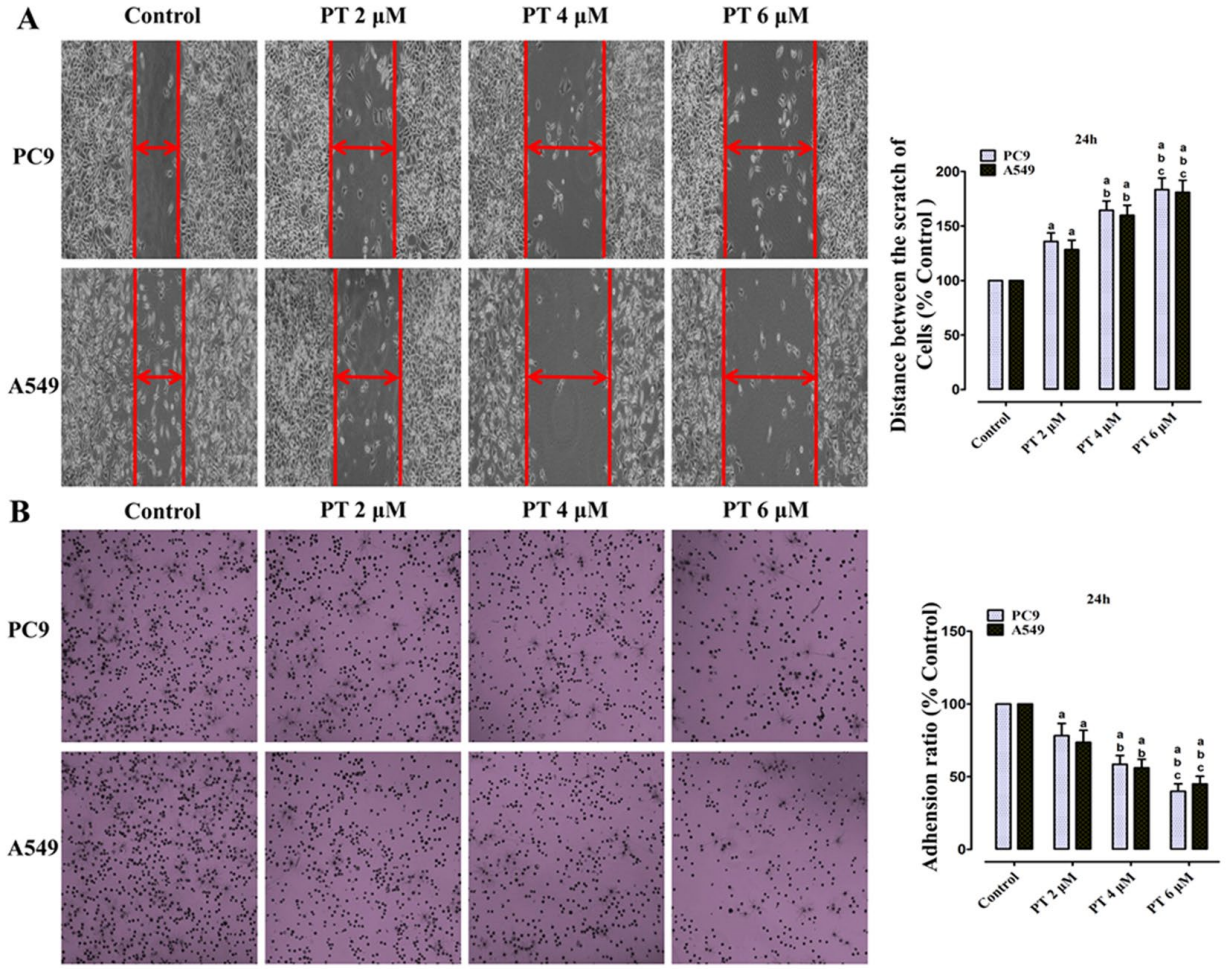
Effect of PT treatment on the abilities of migration and adhesion in PC9 and A549 cells (24 h). (**A**) Representative wound healing images of each cells were shown, and the migratory ability is expressed as the mean distance between the two sides of the scratch. The mean distance in the control group was set as 100%. (**B**) Representative adhesion images of each cell lines were shown, and the adhesion ability of cells is expressed as an adhesion ratio. The number of adherent cells in the control group was set as 100%. All of the results were expressed as the mean ± SD; n = 6. ^a^P < 0.05 *vs*. the control group, ^b^P < 0.05 *vs*. the 2 μM PT-treated group, ^c^P < 0.05 *vs*. the 4 μM PT-treated group.

The migratory and adhesive abilities of those two NSCLC cell lines via using a wound-healing assay, and an adhesion assay. Based on our preliminary experiments, the relatively low concentrations of PT (2, 4, and 6 μM) did not affect the cell viability in human NSCLC cells (Supplementary Figure 3A). So, after incubation with PT (2, 4, and 6 μM) for 24 h, the scratch wound distance is 135.85 ± 7.81%, 164.43 ± 8.52%, and 183.34 ± 10.63% in PC9 cells; and 128.33 ± 8.61%, 159.76 ± 9.30%, and 180.86 ± 11.09% in A549 cells, respectively (P < 0.05, compared with the control group, Fig. 4A). Moreover, in contrast to the control group, the cell adhesion ratio decreased to 78.18 ± 8.50%, 58.49 ± 6.08%, 39.97 ± 5.13% in PC9 cells; and 73.69 ± 8.16%, 56.03 ± 5.99%, 44.98 ± 5.33% in A549 cells, respectively (P < 0.05, compared with the control group, Fig. 4B).

### Activation of ERS signaling, autophagy and apoptosis-related proteins induced by PT on PC9 and A549 cells

**Figure 5:**
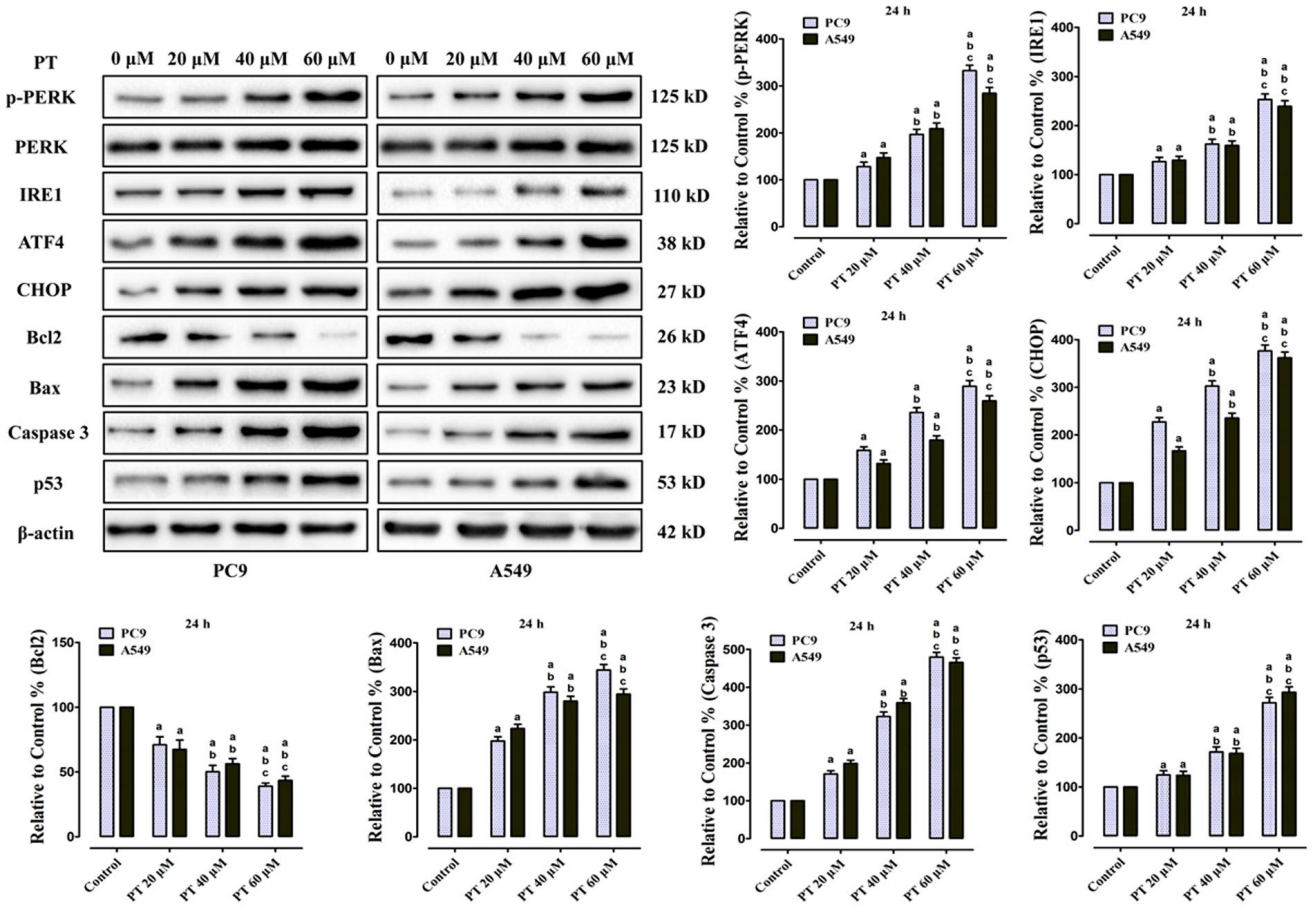
Effect of PT treatment on the ERS signaling and apoptosis-related proteins in PC9 and A549 cells (24 h). Representative western blot results of p-PERK, PERK, IRE1, ATF4, CHOP, Bcl2, Bax, Caspase 3, p53 were shown. Membranes were re-probed for β-actin expression to show that similar amounts of protein were loaded in each lane. All of the results were expressed as the mean ± SD; n = 6. ^a^P < 0.05 *vs*. the control group, ^b^P < 0.05 *vs*. the 20 μM PT-treated group, ^c^P < 0.05 *vs*. the 40 μM PT-treated group.

To investigate the anticancer activity of PT on ERS signaling, ERS-related molecules were detected via western blot in PC9, A549, and HBE cells. Western blot revealed that relative low concentrations of PT treatment (2, 4, and 6 μM) had no effect on the expression of ERS-related proteins in NSCLC cells (P > 0.05, compared with the control group, Supplementary Figure 3A). PT treatment (20, 40, and 60 μM) significantly enhanced the expression of p-PERK, IRE1, ATF4, and CHOP in both NSCLC cell lines in a dose-dependent manner (P < 0.05, compared with the control group, Fig. 5), but the pro-ERS effect on normal bronchial epithelial cells was weaker than on NSCLS cells. PT 20 μM had no effect on ERS signaling upregulation on HBE cells (P > 0.05, compared with the control group, Supplementary Figure 1B), and PT 40 μM slightly promoted ERS signaling expression (P < 0.05). Only PT at higher concentration (60 μM) obviously enhanced ERS signaling expression on HBE cells (Supplementary Figure 1B). Moreover, to investigate whether PT could induce autophagy on NSCLC cells, both western bot and immunocytochemistry analyses revealed PT induced a concentration-dependent accumulation of autophagy-symbolic LC3BII protein in NSCLC cells (Supplementary Figure 4). Furthermore, we also determined whether PT affects the apoptotic proteins in NSCLC cells via measuring the protein expression of Bcl2, Bax, Caspase 3, and p53. PT treatment decreased the expression of Bcl2 and increased the expression of Bax, Caspase 3 and p53 (P < 0.05, compared with the control group, Fig. 5).

### Effect of PT treatment combined with CHOP siRNA on cell viability, Caspase 3 activity, ROS generation, CHOP and apoptosis-related protein levels on PC9 and A549 cells

**Figure 6:**
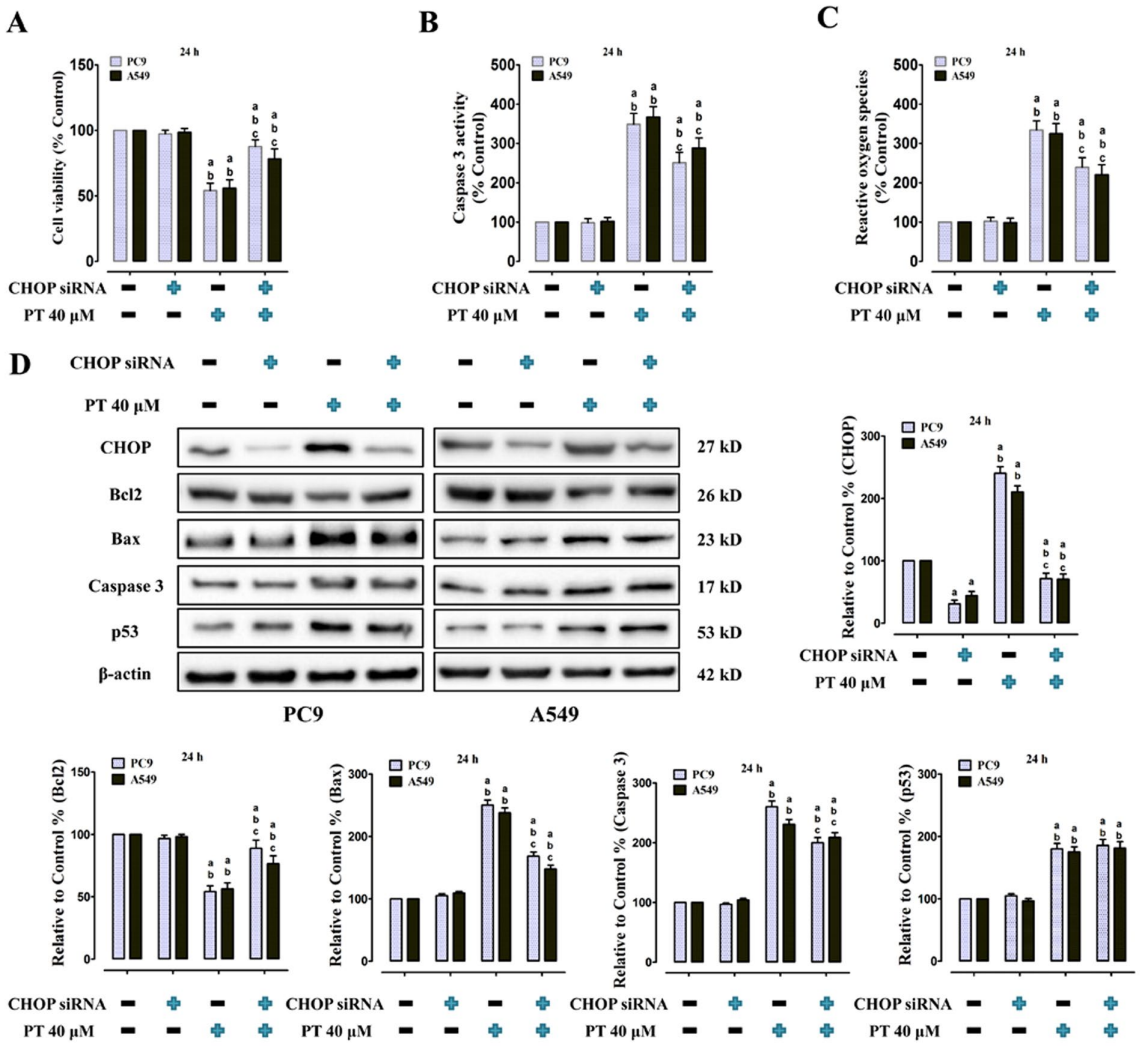
Effect of PT treatment combined with CHOP siRNA on cell viability, Caspase 3 activity, ROS generation, CHOP and apoptosis-related protein levels in PC9 and A549 cells (24 h). (**A**) Cell viability; (**B**) Intracellular Caspase 3 activity; and (**C**) ROS concentration were shown, and those three indexes in the control group were defined as 100%. (**D**) Representative western blot results of the key proteins, including CHOP, Bcl2, Bax, Caspase 3, and p53 were shown. Membranes were re-probed for β-actin expression to show that similar amounts of protein were loaded in each lane. The results were expressed as the mean ± SD; n = 6. ^a^P < 0.05 *vs*. the control group, ^b^P < 0.05 *vs*. the CHOP siRNA-treated group, ^c^P < 0.05 *vs*. the Control siRNA + 40 μM PT-treated group.

A specific CHOP siRNA was used to explore the effect of ERS signaling downregulation on the anti-NSCLC activity of PT treatment *in vitro*. PC9 and A549 cells were first transfected with CHOP siRNA and then treated with PT (40 μM) for an additional 24 h. Transfection with CHOP siRNA significantly decreased the expression of the levels of CHOP in PC9 and A549 cells (P < 0.05, compared to transfection with the control siRNA, Fig. 6D). Moreover, the combination of CHOP siRNA and PT increased cell viability (Fig. 6A), decreased Caspase 3 activity (Fig. 6B) and generation of ROS (Fig. 6C) (P < 0.05, compared with the combination of control siRNA and PT); however, CHOP siRNA alone did not affect cell viability, Caspase 3 activity, and ROS generation compared with the control siRNA (P > 0.05). Moreover, Bcl2 was further upregulated by co-treatment with PT and CHOP siRNA, whereas Bax and Caspase 3 levels were significantly downregulated by co-treatment with CHOP siRNA and PT (P < 0.05, compared with the control siRNA and PT co-treatment, Fig. 6D). Though CHOP siRNA co-treatment decreased PT-induced promotion of Caspase 3 activity and ROS generation, Caspase 3 activity and ROS generation still remained at a high level, indicating CHOP is not the only mediator for apoptosis induction. Interestingly, co-treatment of CHOP siRNA had no effect on PT-induced upregulation of p53 expression (P > 0.05), suggesting p53 upregulation was independent of CHOP activation by PT treatment.

### Effect of PT treatment on Ca^2+^ dyshomeostasis in PC9 cells or combined with CHOP siRNA

The average cytosolic Ca^2+^ concentration in a single cell was determined using the fluorescent probe Fluo-3AM by laser confocal scanning microscopy. PC9 cells were scanned for 3 min to obtain a basal fluorescence intensity level of intracellular Ca^2+^ (F0). Then treated with PT 40 μM and another 12 min under the treatments to obtain the real-time fluorescenceintensity (F). PT 40 μM treatment significantly increased the cytosolic Ca^2+^ level in PC9 cells compared with the control group (P < 0.05, Supplementary Figure 2C), and nearly at 10 min up to the Ca^2+^ peak. Moreover, CHOP siRNA pre-treatment obviously reversed the PT 40 μM induced increase of cytosolic Ca^2+^ level (P < 0.05, compared with PT 40 μM group, Supplementary Figure 2C), with only minor increase of Ca^2+^ level compared with control group (P < 0.05). These results revealed that PT treatment could induce Ca^2+^ dyshomeostasis thus promoting ERS.

### Effect of PT treatment combined with THA on cell viability, Caspase 3 activity, ROS generation, CHOP and apoptosis-related protein levels on PC9 and A549 cells

**Figure 7:**
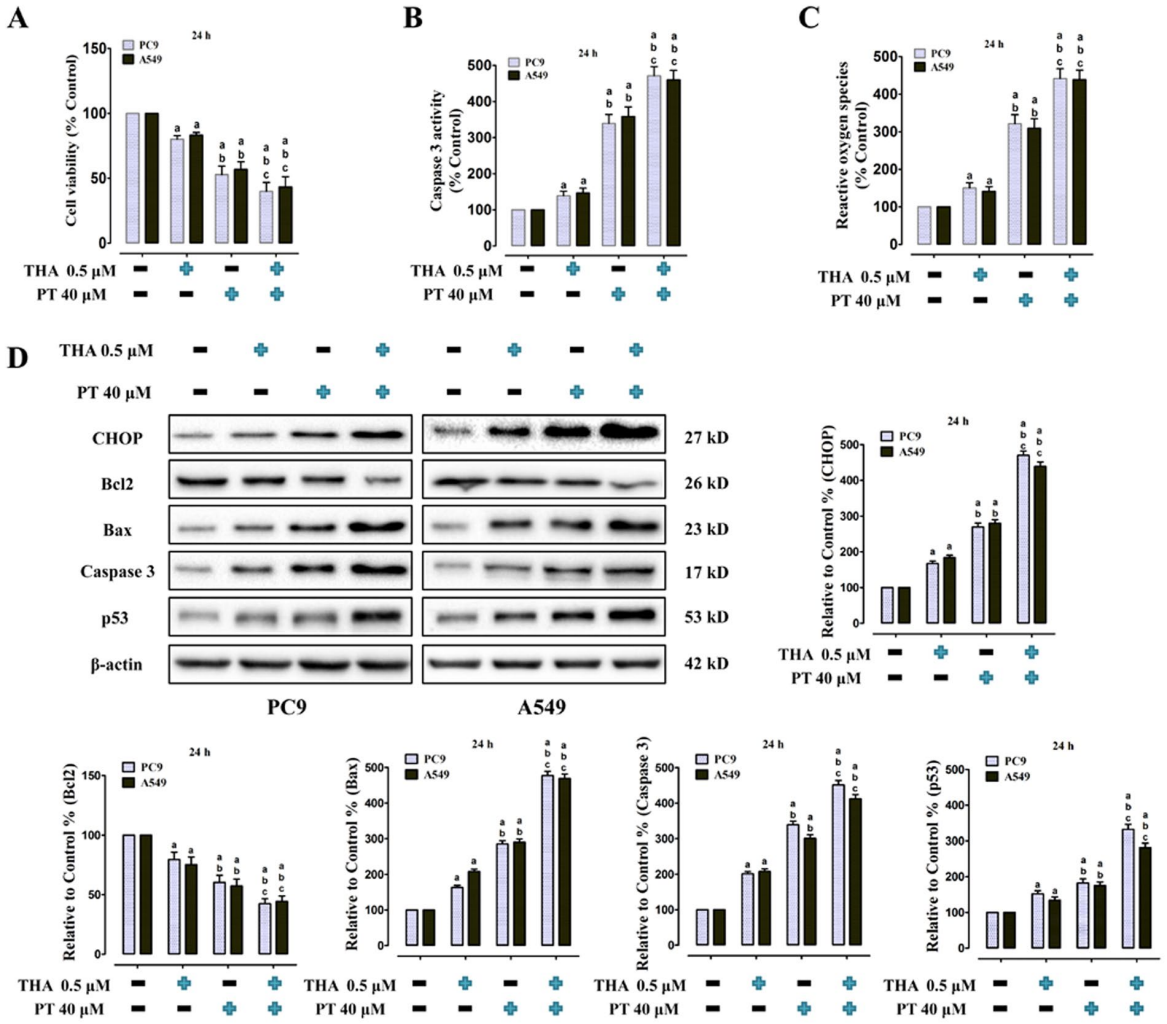
Effect of PT treatment combined with THA on cell viability, Caspase 3 activity, ROS generation, CHOP and apoptosis-related protein levels in PC9 and A549 cells (24 h). (**A**) Cell viability; (**B**) Intracellular Caspase 3 activity; and (**C**) ROS concentration were shown, and those three indexes in the control group were defined as 100%. (**D**) Representative western blot results of the key proteins, including CHOP, Bcl2, Bax, Caspase 3, and p53 were shown. Membranes were re-probed for β-actin expression to show that similar amounts of protein were loaded in each lane. The results were expressed as the mean ± SD; n = 6. ^a^P < 0.05 *vs*. the control group, ^b^P < 0.05 *vs*. the 0.5 μM THA-treated group, ^c^P < 0.05 *vs*. the 40 μM PT-treated group.

THA, one of the classical ERS inducers, causes calcium leakage from ER into the cytosol, thereby exerting anticancer activity by decreasing cancer cell viability through apoptosis. The anti-NSCLC role of THA has also been proven in our previous studies^7^. We combined PT (40 μM) with THA (0.5 μM) and tested their cytotoxicity to PC9 and A549 cells for 24 h. Based on our preliminary experiments, a relatively low concentration and duration of THA treatment (0.5 μM for 24 h) were chosen so that the potential ERS activating effect could emerge more prominently. Compared with the control group, THA treatment alone slightly decreased the cell viability to 82.13 ± 2.69% in PC9 cells and 83.36 ± 2.09% in A549 cells; PT treatment alone decreased the cell viability to 61.81 ± 6.54% in PC9 cells and 58.76 ± 5.96% in A549 cells. However, when PT was combined with THA, the cell viability was significantly decreased to 36.82 ± 3.41% in PC9 cells and 38.23 ± 3.11% in A549 cells. Compared with either PT treatment alone or THA treatment alone, the co-treatment of PT and THA significantly increased Caspase 3 activity (Fig. 7B) and ROS generation (Fig. 7C) (P < 0.05). As shown in Fig. 7D, THA treatment increased CHOP expression in both cell lines (P < 0.05 compared with the control), and PT co-treatment with THA in PC9 and A549 cells significantly upregulated CHOP level compared with either treatment alone (P < 0.05). Moreover, co-treatment of PT and THA also significantly promoted Bax, Caspase 3 and p53 upregulation and downregulated Bcl2 level (P < 0.05 compared with either PT or THA treatment alone), thus the promotion of ERS by THA co-treatment enhanced p53 expression via PT treatment.

### THA co-treatment enhanced inhibition of tumor xenograft growth by PT treatment via promoting ERS signaling and activating apoptosis-related proteins *in vivo*

**Figure 8:**
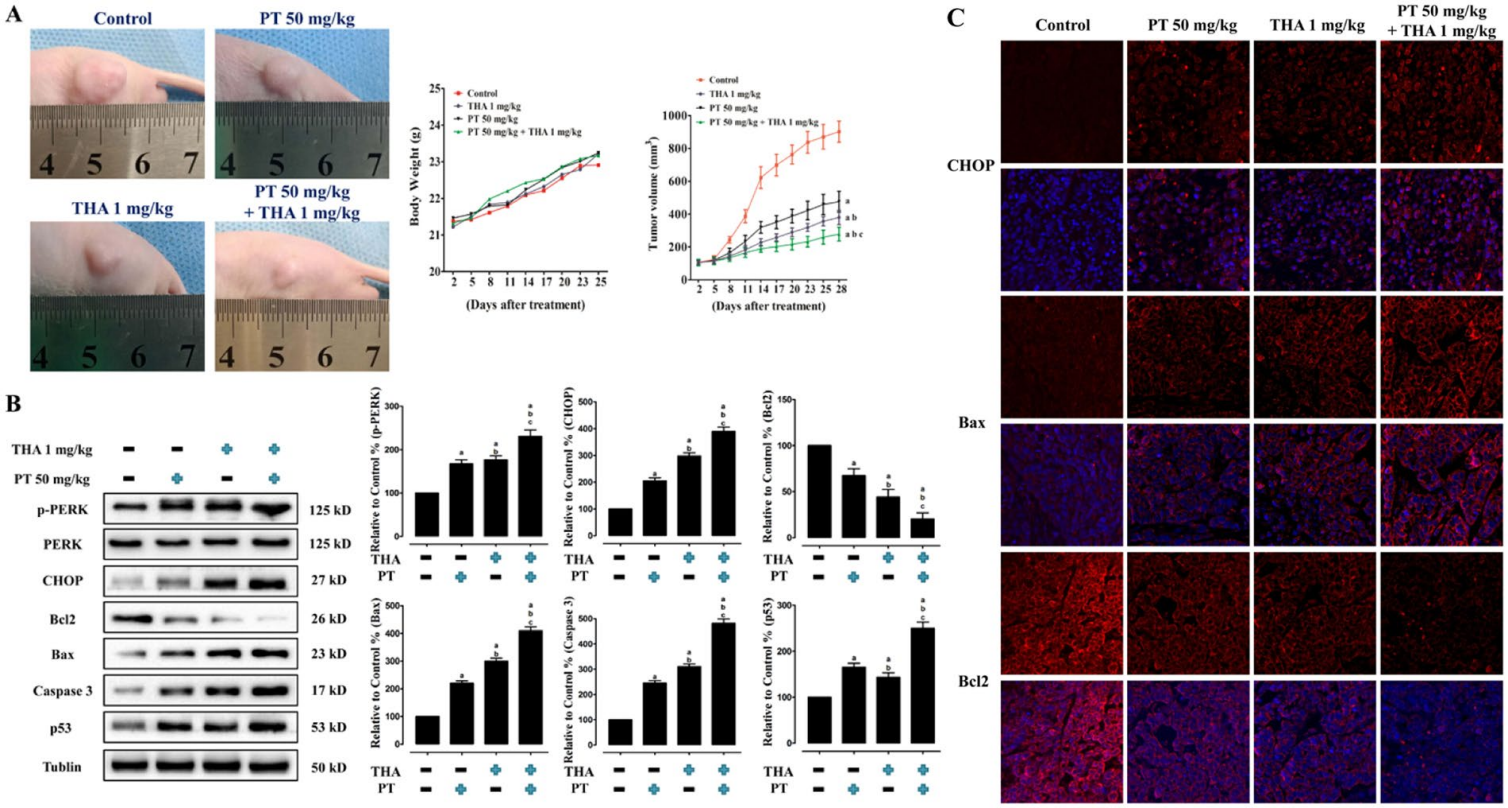
Effect of PT treatment combined with THA on tumor volume, body weight of nude mice, ERS pathways and apoptosis-associated proteins on PC9 tumor xenografts *in vivo*. (**A**) Photographs showing tumor xenograft morphologies in each group, changes in body weight of nude mice, and tumor growth curve drawn from the tumor volumes and treatment duration were provided. (**B**) Representative western blot results of the key proteins, including p-PERK, CHOP, Bcl2, Bax, Caspase 3, and p53 were shown. Membranes were re-probed for Tublin expression to show that similar amounts of protein were loaded in each lane. (**C**) Representative immunohistofluorescence staining images showed CHOP (red fluorescence) in the cell nucleus, Bax and Bcl2 (red fluorescence) in the cytoplasm, and DAPI (dark blue fluorescence) in the cell nucleus. The results were expressed as the mean ± SD; n = 6. ^a^P < 0.05 *vs*. the control group, ^b^P < 0.05 *vs*. the 50 mg/kg PT-treated group, ^c^P < 0.05 *vs*. the 1 mg/kg THA-treated group.

To verify our *in vitro* outcomes and whether PT treatment can inhibit tumor growth *in vivo* studies, we established PC9 xenografts in athymic nude mice and measured their tumor volumes. We found that the nude mice in all of the treatment groups developed subcutaneous tumors, and PT 50 mg/kg treatment or THA 1 mg/kg treatment significantly inhibited tumor growth (P < 0.05, compared with the control group, Fig. 8A), which were consistent with previously reported studies^8, 27^. Further, treatment of THA 1 mg/kg sensitized the anticancer effect of PT 50 mg/kg on tumor growth (P < 0.05, compared with the PT 50 mg/kg or THA 1 mg/kg group), which indicated that PT inhibited the NSCLC cells via activating ERS *in vivo*. Moreover, western blot or immunohistofluorescence analyses showed that PT 50 mg/kg or THA 1 mg/kg treatment promoted the upregulation of ERS signaling molecules (p-PERK and CHOP), and regulation of apoptosis-related proteins (upregulation of Bax, Caspase 3 and p53 levels, and downregulation of Bcl2 protein) (P < 0.05, compared with the control group, Fig. 8B and C), and these effects were significantly enhanced by PT 50 mg/kg and THA 1 mg/kg co-treatment (P < 0.05, compared with the PT 50 mg/kg or THA 1 mg/kg group).

## Discussion

PT, a naturally derived polyphenol compound with multiple properties (e.g., anti-inflammation, anti-obesity, and anti-oxidation), is an analogue of the well-studied resveratrol (RSV), but is reported significantly more bioavailable when ingested^28–30^. Moreover, PT is also a potent anticancer agent against various cancers by different mechanisms^9, 10, 12–18^. Meanwhile, several studies suggested that PT exerts anti-NSCLC activities via the induction of apoptosis-related cell death^8, 21, 27^. However, the actions of PT on NSCLC and the detailed mechanisms responsible for these activities are still not fully elucidated. Consistent with previous studies, PT treatment significantly repressed the cell viability and induced cell death in both PC9 and A549 cell lines with a dose- and time-dependent manner. Furthermore, we found that PT treatment suppressed the migratory and adhesive abilities of NSCLC cells, both of which are major actions associated with tumor metastasis. Additionally, PT treatment obviously inhibited tumor growth in the PC9 xenografts.

During solid cancers progression, cancer cells require a high rate of protein folding and assembly in the ER, but the nutrient requirements eventually exceed the capacity of the existing vascular bed^25^. Therefore, cancer cells usually encounters stressful microenvironments, such as hypoxia and nutrient deprivation, which eventually impairs the generation of ATP and compromises ER protein folding, thus leading to ERS^22, 25^. Meanwhile, UPR is subsequently activated, which aims at restoring ER homeostasis, leading to adaptions, and sustaining cell survival^24, 25^. However, aside from its pro-survival effects, prolonged UPR activation owing to sever or unresolved ERS eventually triggers cell death^22, 23^. Therefore, targeting the ERS signaling activation is a promising anticancer strategy, proved by bortezomib, the first proteasome inhibitor in clinical application for multiple myeloma and Burkiit lymphoma, of which the mechanisms are at least in part via ERS activation^25, 26^. Furthermore, evidence for promoting ERS signaling as the anti-NSCLC therapy, especially activating the death effector CHOP via the PERK-ATF4 cascade, is compelling^7, 31, 32^. In our study, we explored the actions of ERS in the anti-NSCLC activities of PT. ER is the main storage site of Ca^2+^, and any disruption in its accumulation can promote ER stress^23, 33^. Our study found PT treatment could immediately disturb the Ca^2+^ homeostasis in ER and cytoplasm, and significantly upregulate cytosolic Ca^2+^ levels in PC9 cells. Knockdown of CHOP by siRNA *in vitro* thus effectively inhibited the PT-induced Ca^2+^ dyshomeostasis in ER and upregulation of cytosolic Ca^2+^ levels in PC9 cells. Ca^2+^ released from ER has irreversible effects on cell functioning and ultimately leads to apoptotic or necrotic cell death^34^. Consistent with Moon *et al*. work of PT on breast cancer^34^, PT-induced cytosolic Ca^2+^ upregulation may contribute to NSCLC cells apoptosis. Moreover, our results also showed that PT treatment increased the expression of ERS-related proteins (IRE1, p-PERK, ATF4, and CHOP) in PC9 and A549 cells. Additionally, p-PERK and CHOP upregulation were also observed in PC9 cell-based xenografts treated with PT. Knockdown of CHOP by siRNA *in vitro* significantly reduced the anticancer activities of PT. In contrast, the anti-NSCLC effects of PT treatment were enhanced by THA in *vivo* and *in vitro*, one of the classical ERS inducers. THA causes ER calcium depletion through irreversible inhibition of the ER Ca^2+^-ATPase pump which was responsible for the influx of Ca^2+^ from cytosol^33, 35^. Therefore, these outcomes suggest that the anti-NSCLC actions of PT is regulated, at least in part, by the stimulation of ERS signaling pathway.

CHOP is a transcription factor whose induction represses cell proliferation and induces apoptosis via suppressing anti-apoptotic proteins (e.g., Bcl2 and Bcl-xL, Mcl-1) and upregulating pro-apoptotic proteins (e.g., Bax, Bak, Bim), thus connecting ERS and mitochondrial-initiated apoptosis^2, 7, 10, 25^. Examples include inactivation of Bcl2, translocation of Bax and Bak from ER to mitochondrial, enhanced ER Ca^2+^ flux, and caspases activation in response to overwhelmed ERS^2, 7, 10, 22, 36^. In present study, we found PT treatment dose-dependently decreased Bcl2 expression, increased Bax expression, and upregulated Caspsase 3 protein level and activity both *in vivo* and *in vitro*. Furthermore, our *in vivo* studies confirmed that the use of CHOP siRNA reversed the anti-cancer effects mentioned above, whereas treatment with THA thus enhanced those effects. Moreover, pre-incubation with z-DEVD-fmk, a Caspase 3 specific inhibitor, completely inhibited PT-induced increase of Caspase 3 activity and abolished PT-induced cell death in NSCLC cells. These results indicate that PT induces NSCLC cells apoptosis via regulation of Bcl2 family proteins, which are connected with the activation of ERS signaling and Caspase 3-dependent.

The master tumor suppressor p53 is a key protein in preventing cell transformation and tumor progression^14^. Numerous studies have revealed the connection between ERS and p53 signaling pathway^14, 37^. ERS triggers p53-dependent suppression of p21 thus induction of apoptosis^14^. Moreover, instead of functioning as a transcription factor, p53 also locates at the ER and mitochondria-associated membranes, and mediates Ca^2+^ signal-dependent apoptosis during ERS^14^. In present study, we found PT treatment or THA treatment could increase p53 levels in *vitro* and *in vivo*, and co-treatment of PT and THA significantly enhanced this effect, suggesting ERS activation involved in PT-induced increase of p53 protein levels. However, above mentioned results were not interfered by CHOP siRNA, suggesting PT-induced p53 upregulation is CHOP independent, and CHOP is not the only mediator for ERS-related apoptotic induction by PT treatment.

Mitochondrial oxidative stress and the onset of ERS often occur together^38^, and their molecular events linking may attribute to ROS generation^38, 39^. ROS has been identified as a potential anticancer target and its accumulation may represent a cause or a result of prolonged ERS^2, 40, 41^. PERK has been recently reported uniquely enriched at the mitochondria-associated ER membranes^39^. Despite the canonical signaling via the PERK-ATF4-CHOP branch of ERS induces cell apoptosis, Verfaillie and colleagues have demonstrated that PERK at the ER-mitochondria contact sites mediates the ROS-based mitochondrial apoptosis during persistent ERS^39^. In addition, several studies also suggested that activation of CHOP also contributed to ROS generation^13, 23, 42^. In our studies, the suppression of NSCLC cell viability by PT treatment was associated with the rapid increase in ROS generation, and decrease of GSH content and MMP. Moreover, CHOP siRNA treatment attenuated the production of ROS and upregulated the level of GSH, whereas these effects of PT were enhanced by THA co-treatment. GSH is a major non-protein cellular antioxidant that can eliminate intracellular ROS, and the degree of ROS level and perturbations in the GSH redox balance are critical in determining whether cells undergo survival or elimination processes^10, 43, 44^. A recent study by Benlloch *et al*. reported that PT treatment suppressed the anti-oxidative properties of melanoma and pancreatic cancer cells via downregulation of GSH and glucocorticoid levels, and inhibition of glucocorticoid receptor and nuclear factor (erythroid-derived 2)-like 2 (Nrf2)-dependent antioxidant defense system^16^. Based on above results, we propose that the induction of ERS-ROS-dependent cell death signaling may be an important anti-NSCLC mechanism of PT treatment.

Autophagy activation has been proposed as an alternative mechanism of cell death induced by polyphenols^45^. Consistent with other studies^18, 21, 45^, we found PT treatment promoted the induction of autophagy in NSCLC cells. Numerous studies also revealed intensive connections between the activation of ERS and autophagy involved in the induction of apoptotic cell death on cancer cells^46–48^. However, whether the promotion of autophagy by PT treatment antagonizing^18, 21^ or participating^45^ the anti-NSCLC effect of PT remains controversial, thus warranting further investigation.

**Figure 9:**
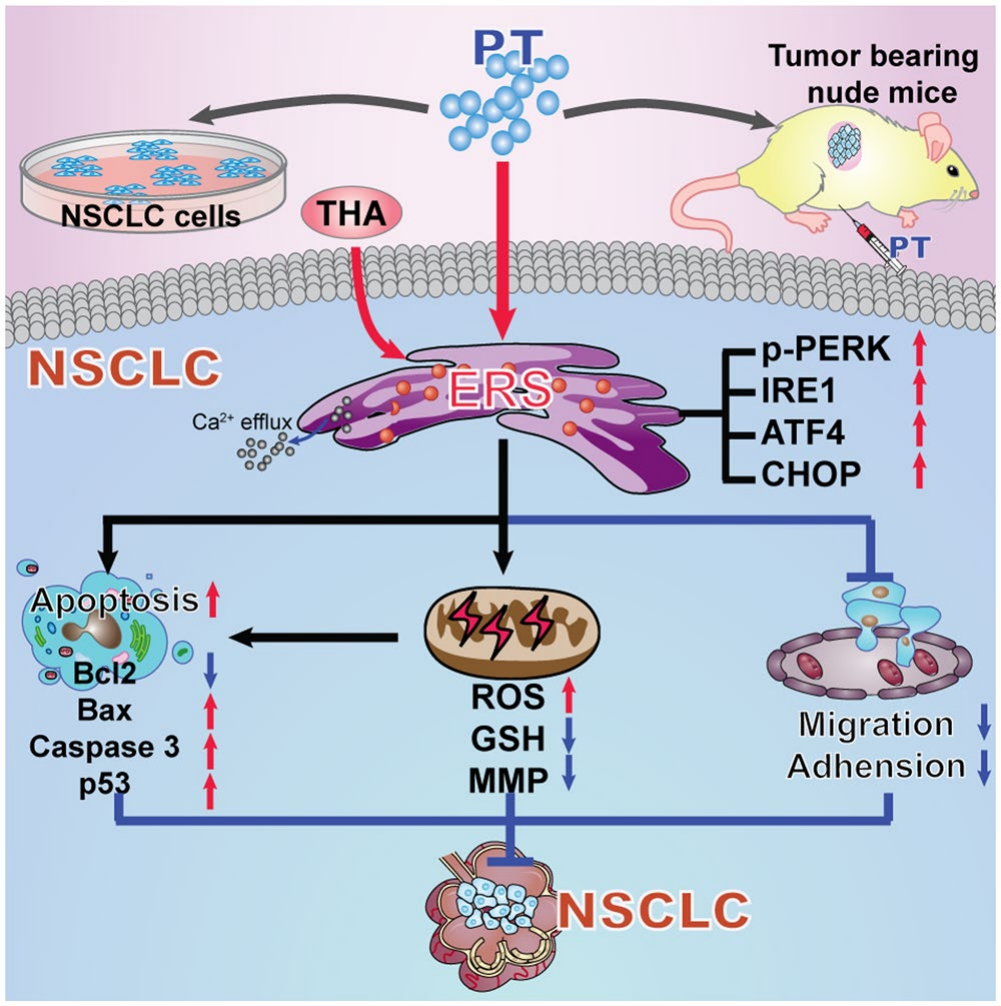
Schematic diagram about the anti-NSCLC activity of PT via the stimulation of ERS. Our *in vivo* and *in vitro* studies showed that PT treatment could stimulate ERS and upregulate a series of ERS-related molecules (i.e., p-PERK, IRE1, ATF4, CHOP) and promoting the efflux of Ca^2+^ from ER into cytoplasm, thus promoting apoptosis (mechanisms via upregulation of Bax, Caspase 3 and p53 expression, and decrease of Bcl2 level), enhancing ROS generation and MMP depolarization, reducing intracellular GSH levels, and inhibiting the ability of migration and adhesion in NSCLC cells. Moreover, THA treatment can enhance the anticancer activity of PT on NSCLC. PT, pterostilbene; THA, thapsigargin; ERS, endoplasmic reticulum stress; NSCLC, non-small-cell lung cancer; p-PERK, phosphorylated-PKR-like ER kinase; IRE1, inositol-requiring enzyme 1; ATF4, activating transcription factor 4; CHOP, C/EBP-homologous protein; Bcl2, B-cell lymphoma-2; Bax, Bcl2-associated X protein; ROS, reactive oxygen species; GSH, glutathione; MMP, mitochondrial membrane potential.

Collectively, our experiments provide *in vivo* and *in vitro* mechanistic evidence that PT is a potent anti-NSCLC agent via activating persistent ERS signaling. Suppression of ERS desensitizes the anticancer effects of PT on NSCLC, whereas enhanced by ERS stimulators. In addition, the PT treatment induced ERS-pro-apoptotic mechanisms on NSCLC may be related to both PERK-ATF4-CHOP cascade, activation of p53, and ERS-ROS-based cell death signaling. Above all, activation of ERS signaling is a promising anticancer therapy for NSCLC (Fig. 9). A small clinic trail with an enrollment of 80 confirmed PT is generally safe for use in humans at doses up to 250 mg per day, and has no direct side-effect on hepatic or renal function^49^. In our studies, compared with NSCLC cells, HBE cells were well tolerated with PT at relatively low concentrations. However, up to the concentration of 60 μM, PT had similar pro-apoptotic and pro-ERS effects on both NSCLC cells and HBE cells, suggesting concentration of PT should be maintained at a relatively effective level for killing cancer cells with no side-effects in clinic. Moreover, PT has also been proven to prevent lung cancer carcinogenesis at a lower concentration (5 μM)^49^, and inhibit the lung cancer stem cells generation and its associated malignancy^20^. Thus, PT displays potent anti-NSCLC properties, and its detailed anticancer mechanisms and application in clinic still warrant further investigation.

## Materials and Methods

### Drugs and reagents

PT, thapsigargin (THA, one of the ERS inducers), dimethyl sulfoxide (DMSO), 4′,6- diamidino-2-phenylindole (DAPI), and 2′,7′-dichlorofluorescein diacetate (DCFH-DA), 3-(4,5-dimethylthiazol-2-yl)-2,5-diphenyltetrazolium bromide (MTT), were purchased from Sigma-Aldrich (St. Louis, MO, USA). The following antibodies were used: anti-IRE1, anti-Bcl2, anti-Bax, anti-Caspase 3, anti-p53, anti-β-actin, and anti-Tublin (Cell Signaling Technology, Beverly, MA, USA); anti-LC3B (Abcam, Cambridge, MA, UK); anti-ATF4, anti-p-PERK, anti-PERK, and anti-CHOP (Santa Cruz Biotechnology, Santa Cruz, CA, USA); goat anti-rabbit, goat anti-mouse, and rabbit anti-goat secondary antibodies (Zhongshan Company, Beijing, China). The Cell Counting Kit-8 (CCK-8) was purchased from Dojindo (Kumamoto, Japan). Terminal deoxynucleotidyl transferase dUTP nick-end labeling (TUNEL) and Annexin V-FITC/PI Apoptosis kits were both purchased from Roche Diagnostics (Mannheim, Germany). The fluorescent dye JC-1, reactive oxygen species (ROS) assay kit, Fluo-3AM, Pluronic F-127 and goat serum were purchased from the Beyotime Institute of Biotechnology (Nanjing, Jiangsu, China). Caspase 3 colorimetric assay kit were purchased from R&D Systems (Minneapolis, MN, USA). Benzyloxycarbonyl-Asp (OMe)-Glu (OMe)-Val-Asp (OMe)-fluoromethylketone (z-DEVD-fmk) was purchased from BD Biosciences (San Diego, CA, USA). The glutathione (GSH) assay kit was obtained from Shanghai Enzyme-linked Biotechnology Co., Ltd. (Shanghai, China). The Cy3, goat anti-rabbit IgG was purchased from Abbkine (California, USA).

### Cell culture and siRNA transfection

Human NSCLC cells (PC9 and A549 cells) were provided by the cell bank of type culture collection of Chinese academy of sciences (Shanghai Institute of Cell Biology, Chinese Academy of Sciences, Shanghai, China). Human bronchial epithelium cells (HBE cells) were purchased from the American Tissues Culture Collection (ATCC, Rockville, MD, USA). The NSCLC and HBE cells were routinely cultured in Dulbecco’s modified Eagle’s medium (DMEM, Gibco, Grand Island, NY, USA), supplemented with 10% fetal bovine serum (FBS, Gibco), L-glutamine (2 mM), penicillin (100 units/ml), and streptomycin (100 units/ml) (Invitrogen, Breda, Netherlands) according to the provider’s instructions. The three kinds of cell were than incubated at 37 °C in a humidified atmosphere containing 5% CO_2_.

The human CHOP siRNA and nonsilencing were chemically synthesized by Genepharma (Shanghai, China). PC9 and A459 cells were respectively plated on 6-well plates at 1 × 10^5^ cells per well in 2 mL of antibiotic-free normal growth medium supplemented with FBS. After the cells reached 60–70% confluence, then were transfected with scrambled siRNA or CHOP-targeted siRNA duplex (100 pmol/L) using Lipofectamine 2000 (Invitrogen) as the manufacturer’s recommendations. Cells were harvested after 48 h transfection and prepared for further experiments.

### Cell treatment

PT stock solution was prepared in DMSO and diluted in culture media immediately prior to an experiment. FBS-free DMEM with same volume of DMSO (0.1%) was used as the control. The PC9, A549, and HBE cells were treated with PT (20 μM, 40 μM, 60 μM) in our first part of experiments, and then treated with PT (40 μM) in the absence or presence of THA (0.5 μM) and CHOP siRNA for different periods of time in the second part of our experiments on NSCLC cells. After those treatments, cells were harvested for further analysis.

### Analysis of cell viability

CCK-8 was used to measure cell viability according to the manufacturer’s instructions. PC9, A549, and HBE cells were seeded in the 96-well plate; five parallel replicates were prepared and then were exerted various treatments, and the control group was treated with DMEM and DMSO (0.1%). Next, CCK-8 (10 μL) was added in each well then incubated at 37 °C in a 5% CO_2_/95% O_2_ humidified incubator for 2 h. Optical density (OD) values were obtained at 450 nm using a microplate reader (SpectraMax 190, Molecular Device, USA), and cell viability was expressed as the OD value, and images were taken using a 600D camera (Canon Company, Japan). All experiments were repeated three times.

### Analyses of cell apoptosis by TUNEL and Annexin V-FITC/PI assay

The levels of cellular apoptosis in PC9, A549 and HBE cells were analyzed via a TUNEL assay as previously described^2^, using an *in situ* cell death detection kit according to the manufacturer’s directions. The TUNEL assay was performed to stain the nuclei of the apoptotic cells (*green*), and DAPI was used to stain the nuclei of all cells (*blue*). Images were obtained by using an Olympus FV1000 confocal microscope (Olympus, Japan) laser confocal microscope, and the TUNEL-positive cells were counted in 5 randomly selected fields under high-power magnification. The apoptotic index was expressed as the ratio between the number of TUNEL-positive cells and the total number of DAPI-positive cells counted × 100%.

For AnnexinV/PI double staining, cells were cultured in confocal dishes for 24 h. After treatments, cells were incubated in binding buffer containing Annexin V-FITC and propidium iodide (PI) at room temperature for 10 min. Annexin V-FITC was performed to stain the membrane of the early-stage apoptotic cells (green), and PI was used to stain the nuclei of late apoptotic or necrotic cells (red). Images were obtained by using an Olympus FV1000 confocal microscope (Olympus, Japan) laser confocal microscope, and the only Annexin V-positive cells were counted in 5 randomly selected fields under high-power magnification. The apoptotic index was expressed as the ratio between the number of Annexin V-positive cells and the total number of cells counted × 100%.

### Analysis of cell mitochondrial membrane potential

The changes in mitochondrial membrane potential (MMP) was reflected by the cells staining with the fluorescent dye JC-1 as previously described^8^. Briefly, lung cancer cells (2 × 10^5^ cells) were cultured in confocal dishes. After different treatments, cells were incubated with JC-1 working solution in the dark at 37 °C for 20 min. Cells exhibiting red fluorescence are in normal state with high MMP, however, when the cells are in an apoptotic or necrotic state, the JC-1 is present as a monomer and MMP is decreased, thus the dye emits green fluorescence. Images were obtained by using an Olympus FV1000 (Olympus, Japan) laser confocal microscope, and the results were expressed as the proportion of cells with a low MMP.

### Analyses of Caspase 3 activity, ROS generation, and GSH level

After treatment, Caspase 3 activity was measured via using a colorimetric assay kit according to the manufacturer’s instructions. The cells or tissue lysates (20 μl) were added to a buffer containing a p-nitroaniline (pNA)-conjugated substrate for Caspase 3 (Ac-DEVD-pNA) to yield a 100 μl reaction volume. The reactions were performed for 2 h at 37 °C. The released pNA concentrations were calculated based on the absorbance values at 405 nm and the calibration curve of the defined pNA solutions. The Caspase 3 activity in the control group was set as 100%.

To analyze intracellualr ROS generation, samples were trypsinized and subsequently incubated in DCFH-DA (5 μM) in PBS for 2 h at 37 °C. After incubation, the DCFH fluorescence of samples were measured using an FLX 800 fluorescence microplate reader at an excitation of 488 nm and an emission of 522 nm (Biotech Instruments, Inc., USA). A cell-free condition was used as the background, and the fluorescence intensity in the control group was defined as 100%. The generation of intracellular reduced GSH was simultaneously measured using commercial kits according to the provided instructions and as previously described^2^. Fluorescent products were measured using an FLX 800 fluorescence microplate reader at an excitation of 335 nm and an emission of 541 nm. The GSH level in the control group was set as 100%.

### Analyses of cell migration and adhesion

We performed adhesion and migration analyses at lower concentrations of PT in order to do not affect cell activity. As our preliminary experiments showed that PT (lower than 6 μM) treatment for 24 h did not affect the proliferation of both PC9 and A549 cells. A cell culture wound-healing assay was performed to analyze cell migration, and 1 × 10^5^ cells were seeded in the 6 well plate in basal medium containing 10% FBS at 37 °C with a humidified 5% CO_2_ in incubator. When the cells were grown to confluence, a linear wound was made in the confluent monolayer using the 200 uL micropipette tip. Then cells were washed with PBS to remove the cellular debris. After treatment with PT (2 μM, 4 μM, and 6 μM) for 24 h, the movements of the wound edges were monitored under the microscope. The results are showed as the distance between the cells on either side of the scratch.

After treatment, 100 μL of medium containing 1 × 10^4^ cells were incubated to each well in a 96-well plate for 30 min at 37 °C. The medium in each well was then discarded for adhesion analysis. The adherent cells were stained with MTT. The stained cells were observed using an inverted phase-contrast microscope. Next, images were obtained via the 600D camera (Canon, Japan), and five fields were randomly selected for quantification. Finally, 100 μL of DMSO was added to each well, and the plates were incubated for 30 min at 37 °C with shaking. The OD value of each well at 570 nm was measured using a SpectraMax 190 spectrophotometer (Molecular Devices, Sunnyvale, CA, USA), and the OD value of the control group was normalized to 100%.

### Analyses of cytosolic calcium

Free cytosolic Ca^2+^ was measured by fluorescence imaging using the Ca^2+^ indicator dye Fluo-3AM. PC9 cells were cultured in confocal dishes for 24 h. Cells were washed 3 times with Hank’s balanced salt solution (HBSS) without Ca^2+^ and Mg^2+^, then cells were incubated with 5 μM Fluo-3AM and 0.2% pluronic F-127 in HBSS for 1 h at 37 °C. Afterwards, Fluo-3AM containing solution was removed and cells were incubated for an additional 30 min in HBSS at 37 °C. Before exposed to PT 40 μM (HBSS was applied for control group), the cells were scanned for 3 min to obtain a basal fluorescence intensity level of intracellular Ca^2+^ (F0) and another 12 min under the treatments to obtain the real-time fluorescenceintensity (F), the ratio was F/F0. Fluorescence was excited at 506 nm, emission was detected at 526 nm, and the image was recorded every 30 s by Olympus FV1000 (Olympus, Japan) laser confocal microscope. The results were expressed as the ratio of F/F0.

### Western blot

Western blot procedures were presented as previously described^2^. Briefly, the cells or tumor samples were lysed in sample buffer [150 mM Tris (pH 6.8), 8 M urea, 50 mM DTT, 2% sodium dodecyl sulfate, 15% sucrose, 2 mM EDTA, 0.01% bromophenol blue, and 1% protease and phosphatase inhibitor cocktail], sonicated, boiled, fractionated by SDS-PAGE, transferred to PVDF membranes, and subjected to western blot analysis with various antibodies. The fluorescent signal was detected using a BioRad imaging system (BioRad, Hercules, CA, USA), and the signal was quantified using Image Lab Software (BioRad, Hercules, CA, USA).

### Immunocytofluorescence and immunohistofluorescence staining

NSCLC Cells were cultured in confocal dishes for further treatments. The formalin-fixed paraffin-embedded tumor tissue sections were performed by deparaffinizing and rehydrating the tissues followed by antigen retrieval. Cells or tissue sections were successively blocked with 0.1% Triton X-100 for 15 min, blocked with goat serum for 2 h at room temperature, then incubated with primary antibody overnight at 4 °C. They were washed with PBS and incubated with Cy3, goat anti-rabbit IgG for 2 h at 37 °C. After washed with PBS, they stained with DAPI for 15 min at 37 °C. Images were acquired using Olympus FV1000 confocal microscope (Olympus, Japan).

### Anticancer activity of PT on a xenograft model

Male athymic nude mice were purchased from the Laboratory Animal Centre of the Fourth Military Medical University. The mice were fed and maintained under specific pathogen-free conditions in facilities approved by the American Association for Accreditation of Laboratory Animal Care and in accordance with the current regulations and standards of the United States Department of Agriculture and the United States Department of Health and Human Services. All experiments of animals were carried out in accordance with the Institutional Animal Care and Use Committee guidelines and were approved by the Institutional Animal Ethics Committee of the Fourth Military Medical University (Permit No. 16001, 16002). Nude mice were s.c. injected with 7 × 10^6^ cells transduced with PC9 cells into both flanks of each animal, respectively. The body weight and tumor size of each mouse were measured every 3 days. The estimated tumor volume was calculated using the formula: volume = 0.5 × length × width^2^. Based on the data from a preliminary study, we initiated treatment when V reached approximately 100 mm^3^. Then the mice were randomly allocated to 4 groups (n = 6/group): the control group (0.05% DMSO), PT 50 mg/kg, THA 1 mg/kg, and PT 50 mg/kg and THA 1 mg/kg co-treatment groups. PT and THA were diluted with DMSO in saline and was administered intraperitoneally every day. On day 28, the tumors were excised from euthanized mice for additional analysis.

### Statistical analyses

All of the data are presented as the means ± standard deviation (m ± SD). Between-group comparisons, one-way ANOVA followed by Bonferroni post hoc test was performed with SPSS 13.0 (SPSS Inc., Chicago, USA) software. A P value of less than 0.05 was considered to be significant.

## Electronic supplementary material

Supplementary Information
