# Supplementary material for: Pterostilbene exerts anticancer activity on non-small-cell lung cancer via activating endoplasmic reticulum stress
Source: Sci Rep. 2017 Aug 14;7:8091. doi: 10.1038/s41598-017-08547-0 (PMC5556085; doi:10.1038/s41598-017-08547-0)

Supplementary Information

Title of manuscript:

Pterostilbene exerts anticancer activity on non-small-cell lung cancer via activating endoplasmic reticulum stress

Authors:

Zhiqiang Ma, Yang Yang, Shouyin Di, Xiao Feng, Dong Liu, Shuai Jiang, Wei Hu, Zhigang Qin, Yue Li, Jianjun Lv, Chongxi Fan, Xiaolong Yan and Xiaofei Li

Supplementary information includes:

Supplementary Figures S1-S8

Supplementary Figure 1. Effect of PT treatment on cell viability, apoptosis, ERS-related proteins in HBE cells (24 h). After PT treatment on HBE cells for 24 h, **(A)** Cell morphology was observed under a phase contrast microscope (x200); Cell viability and apoptosis index were presented. **(B)** Representative western blot results of p-PERK, PERK, IRE1, ATF4, CHOP were shown. Membranes were re-probed for β-actin expression to show that similar amounts of protein were loaded in each lane. All of the results were expressed as the mean ± SD; n = 6. [a]P < 0.05 vs. the control group, [b]P < 0.05 vs. the 20 μM PT-treated group, [c]P < 0.05 vs. the 40 μM PT-treated group.

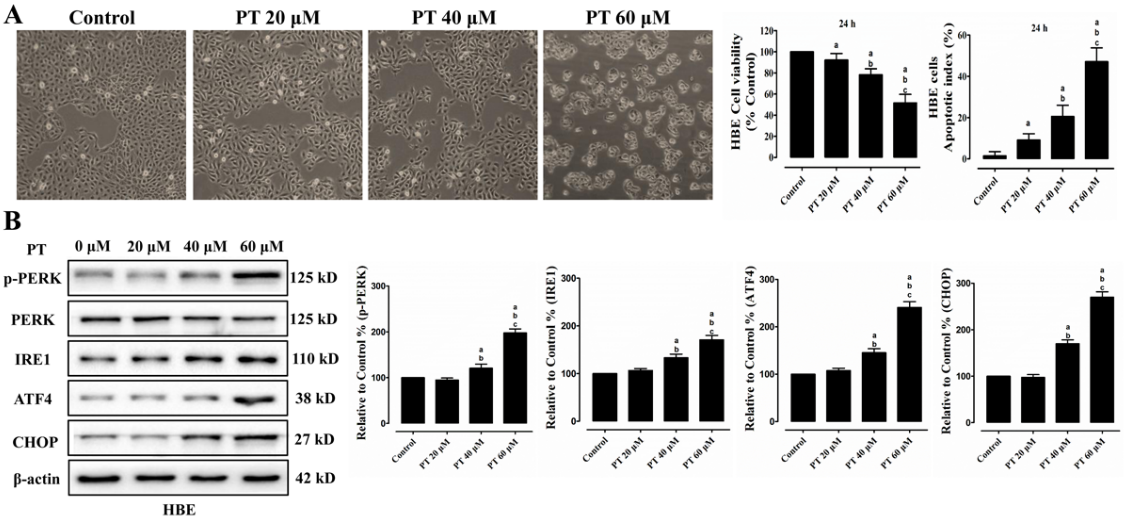

Supplementary Figure 2. Effect of PT treatment on the apoptosis and cytosolic calcium homeostasis, PT treatment combined with z-DEVD-fmk on cell viability and Caspase 3 activity in NSCLC cells. After PT treatment on NSCLC cells for 24 h, **(A)** representative merged images of Annexin V-FITC (green, stained on the membrane, the early-stage apoptotic cells) and PI (red, stained on the nuclei, late apoptotic or necrotic cells with white arrows), and the apoptotic

index were expressed as the proportion of Annexin V-positive cells and the total number of cells. All of the results were expressed as the mean ± SD; n = 6. ^{a}P < 0.05 vs. the control group, ^{b}P < 0.05 vs. the 20 μM PT-treated group, ^{c}P < 0.05 vs. the 40 μM PT-treated group. **(B)** Cell viability and Intracellular Caspase 3 activity were shown, and both indexes in the control group were defined as 100%. The results were expressed as the mean ± SD; n = 6. ^{a}P < 0.05 *vs.* the control group, ^{b}P < 0.05 *vs.* the 30 μM z-DEVD-fmk-treated group, ^{c}P < 0.05 *vs.* the 40 μM PT-treated group. **(C)** Representative trace of 40 μM PT-induced cytosolic Ca^{2+} change for 12min, measured using Fluo-3AM, and a series of screenshot of one cell in the groups were presented. ^{a}P < 0.05 *vs.* the control group, ^{b}P < 0.05 *vs.* the PT 40 μM+CHOP siRNA-treated group.

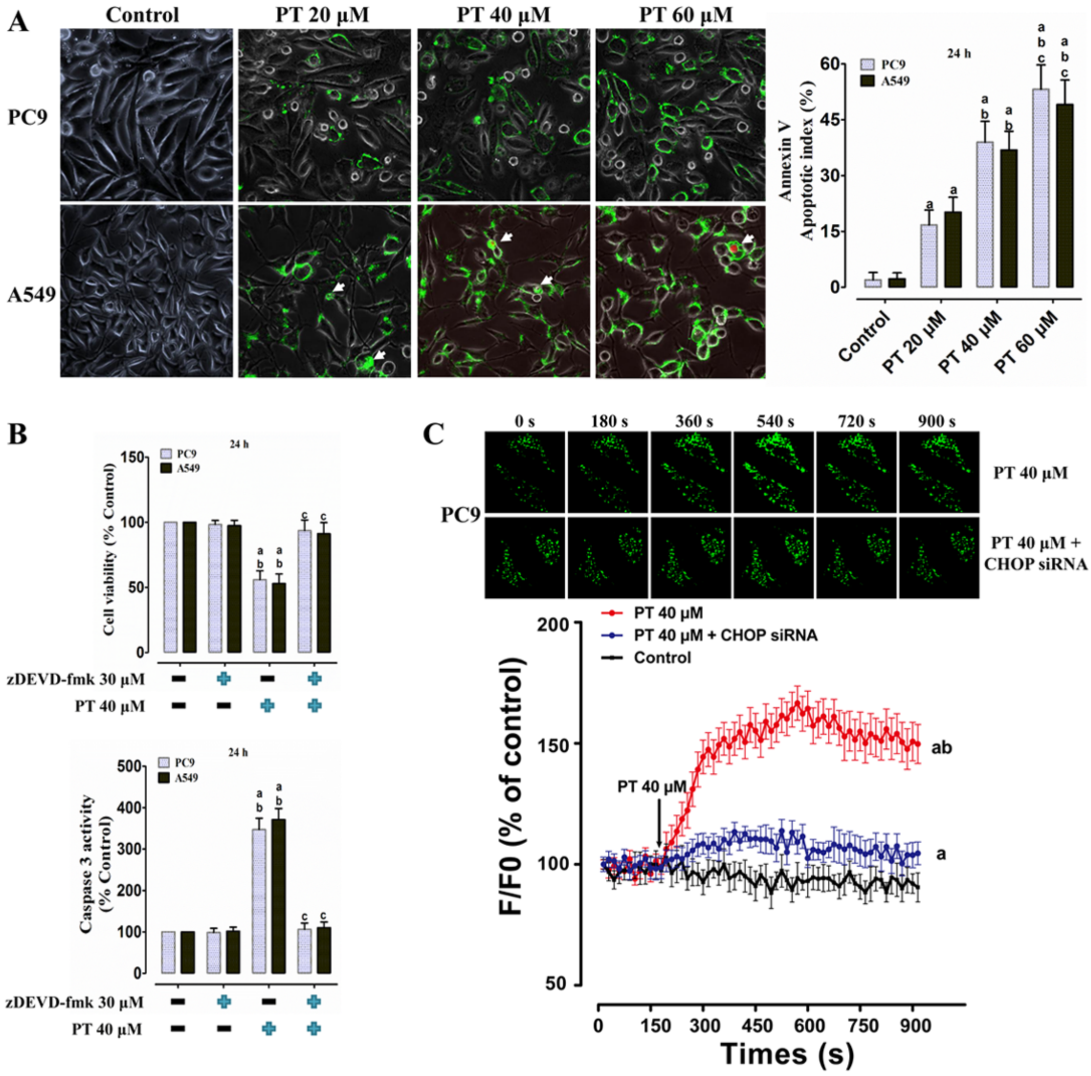

Supplementary Figure 3. Effect of PT treatment with low concentrations on the viability and ERS-related proteins of NSCLC cells. After PT treatment with low concentrations (2, 4, and 6 μM) on NSCLC cells for 24 h, **(A)** Cell viability; **(B)** Representative western blot results of p-PERK, PERK, ATF4, CHOP were shown. Membranes were re-probed for β-actin expression to show that similar amounts of protein were loaded in each lane. All of the results were expressed as the mean ± SD; n = 6.

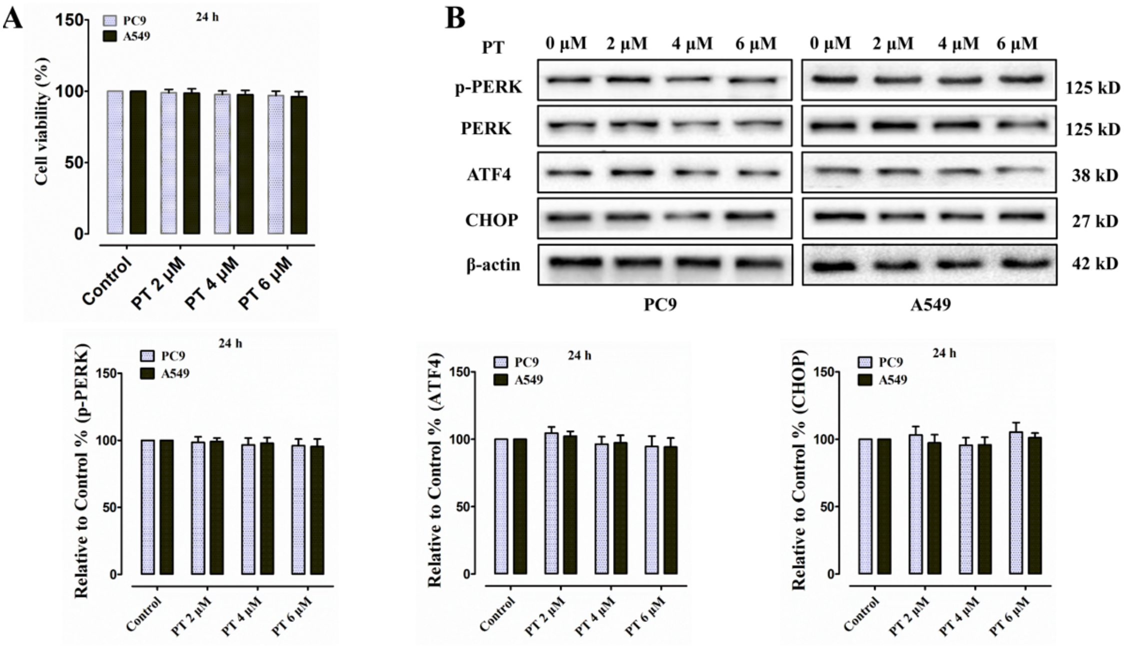

Supplementary Figure 4. Effect of PT treatment on the Autophagy-related LC3II protein in NSCLC cells (24 h). (A) Representative western blot results of LC3BII were shown. Membranes were re-probed for β-actin expression to show that similar amounts of protein were loaded in each lane. All of the results were expressed as the mean ± SD; n = 6. [a]P < 0.05 *vs.* the control group, [b]P < 0.05 *vs.* the 20 μM PT-treated group, [c]P < 0.05 *vs.* the 40 μM PT-treated group. **(B)** Representative immunocytofluorescence staining images showed LC3B (red fluorescence) in the cytoplasm and DAPI (blue fluorescence) in the cell nucleus.

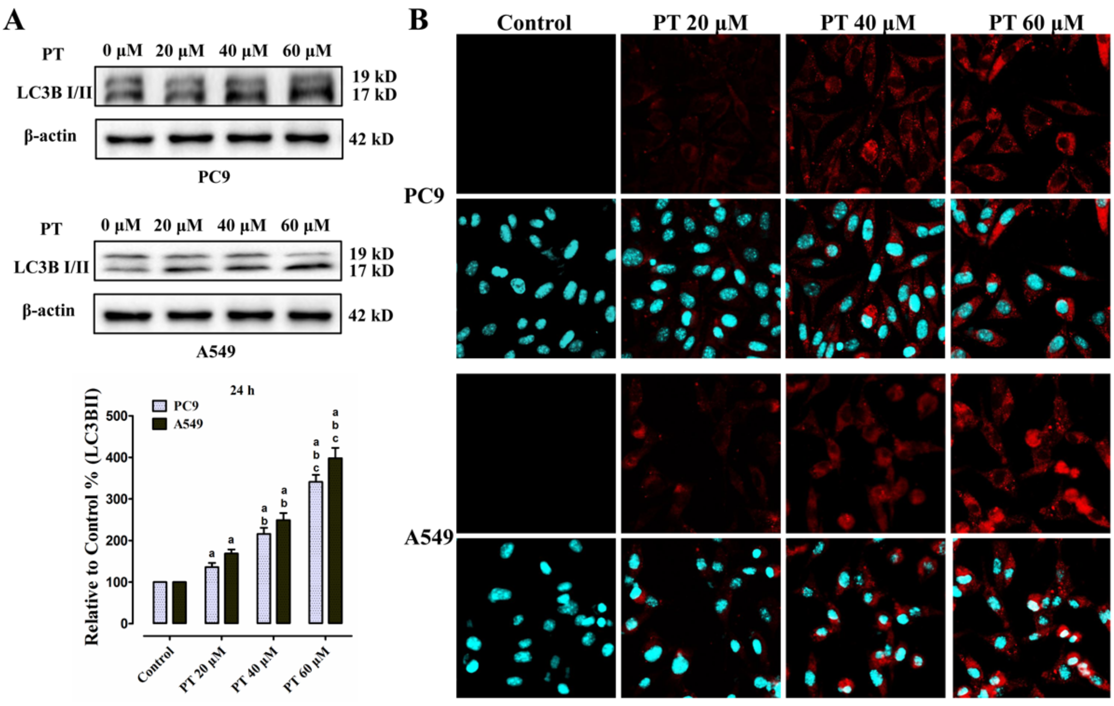

Supplementary Figure 5: The full length blots in Fig. 5

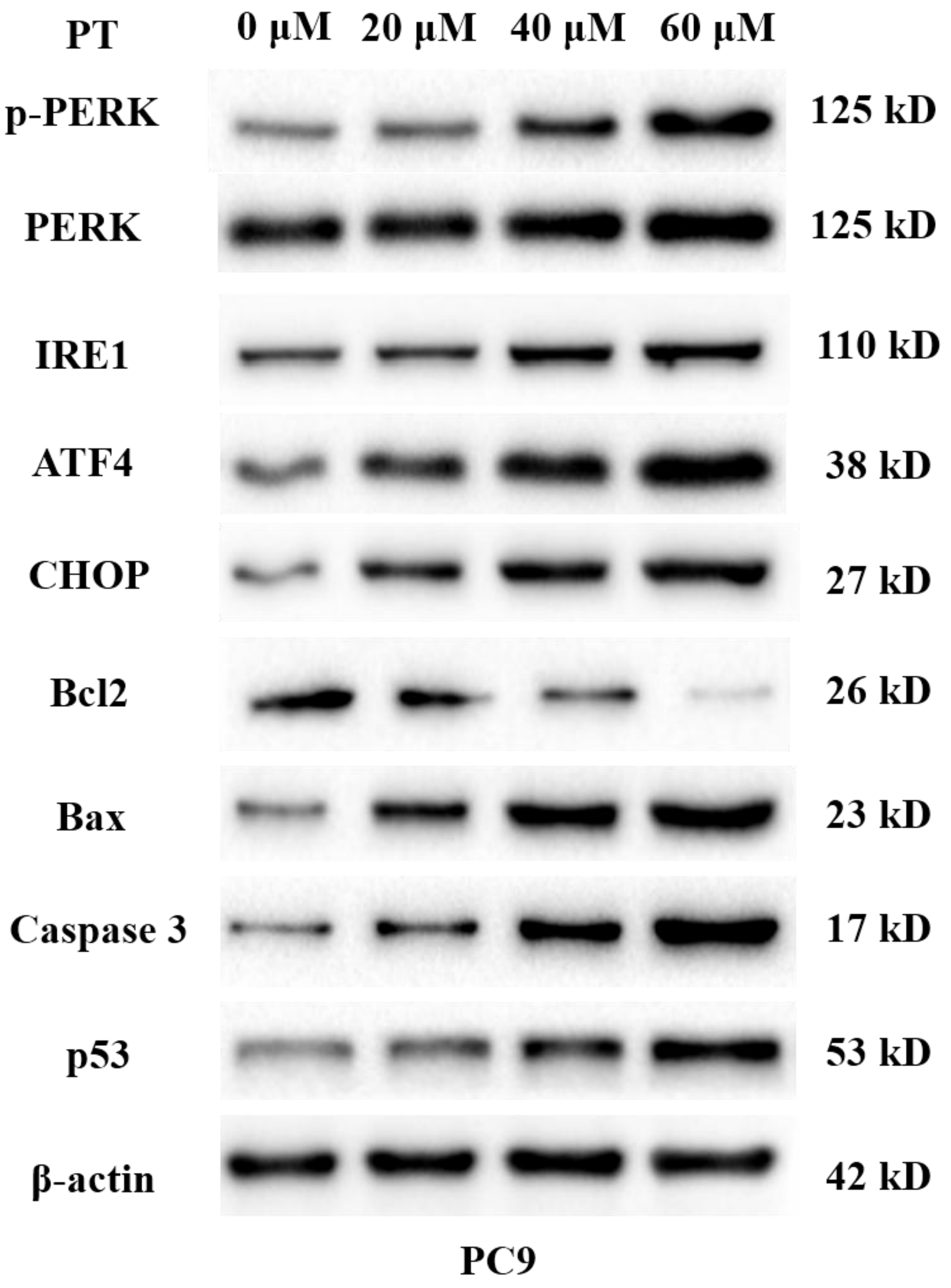

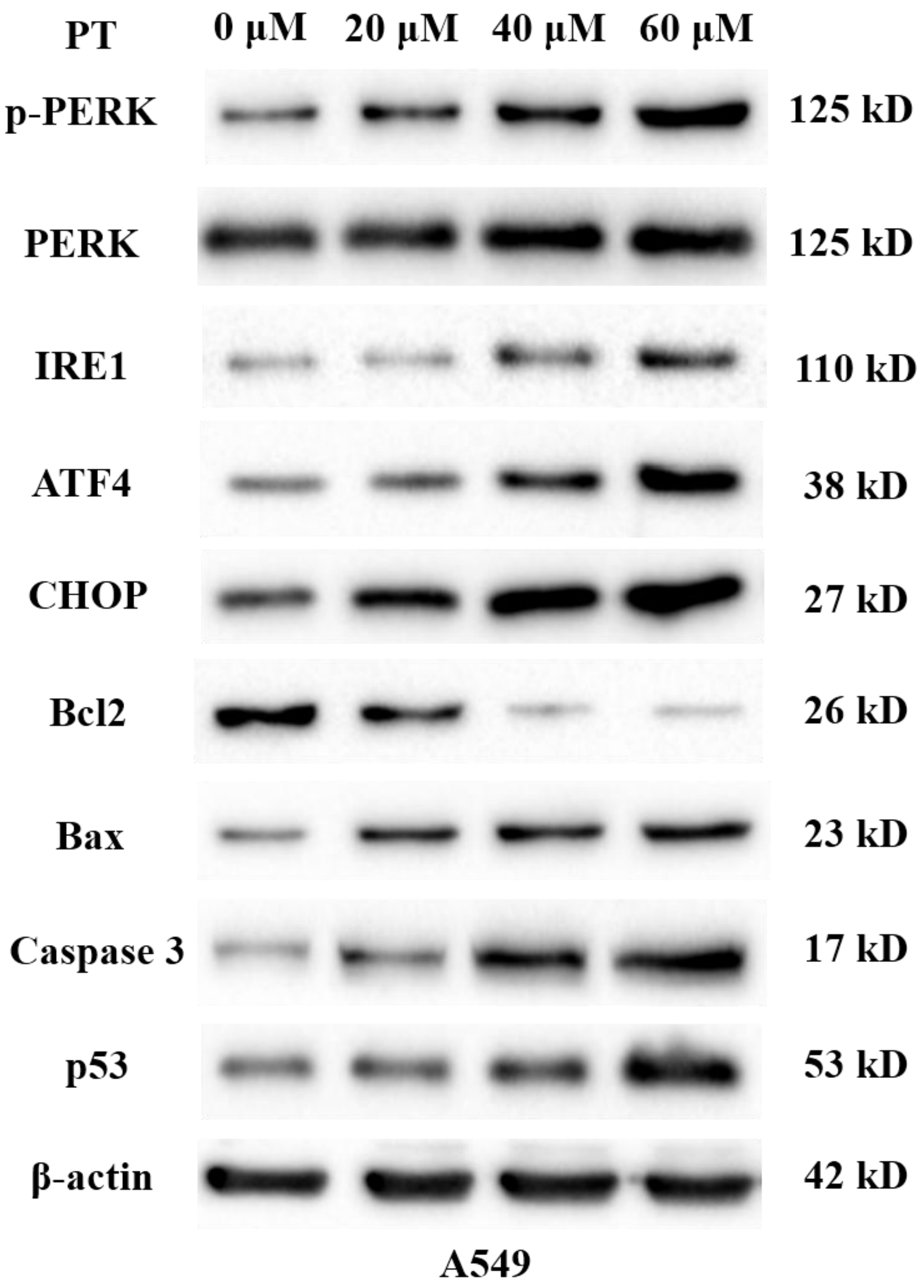
PT
0 μM
20 μM
40 μM
60 μM
p-PERK
125 kD
PERK
125 kD
IRE1
110 kD
ATF4
38 kD
CHOP
27 kD
Bcl2
26 kD
Bax
23 kD
Caspase 3
17 kD
p53
53 kD
β-actin
42 kD
A549

Supplementary Figure 6: The full length blots in Fig. 6D

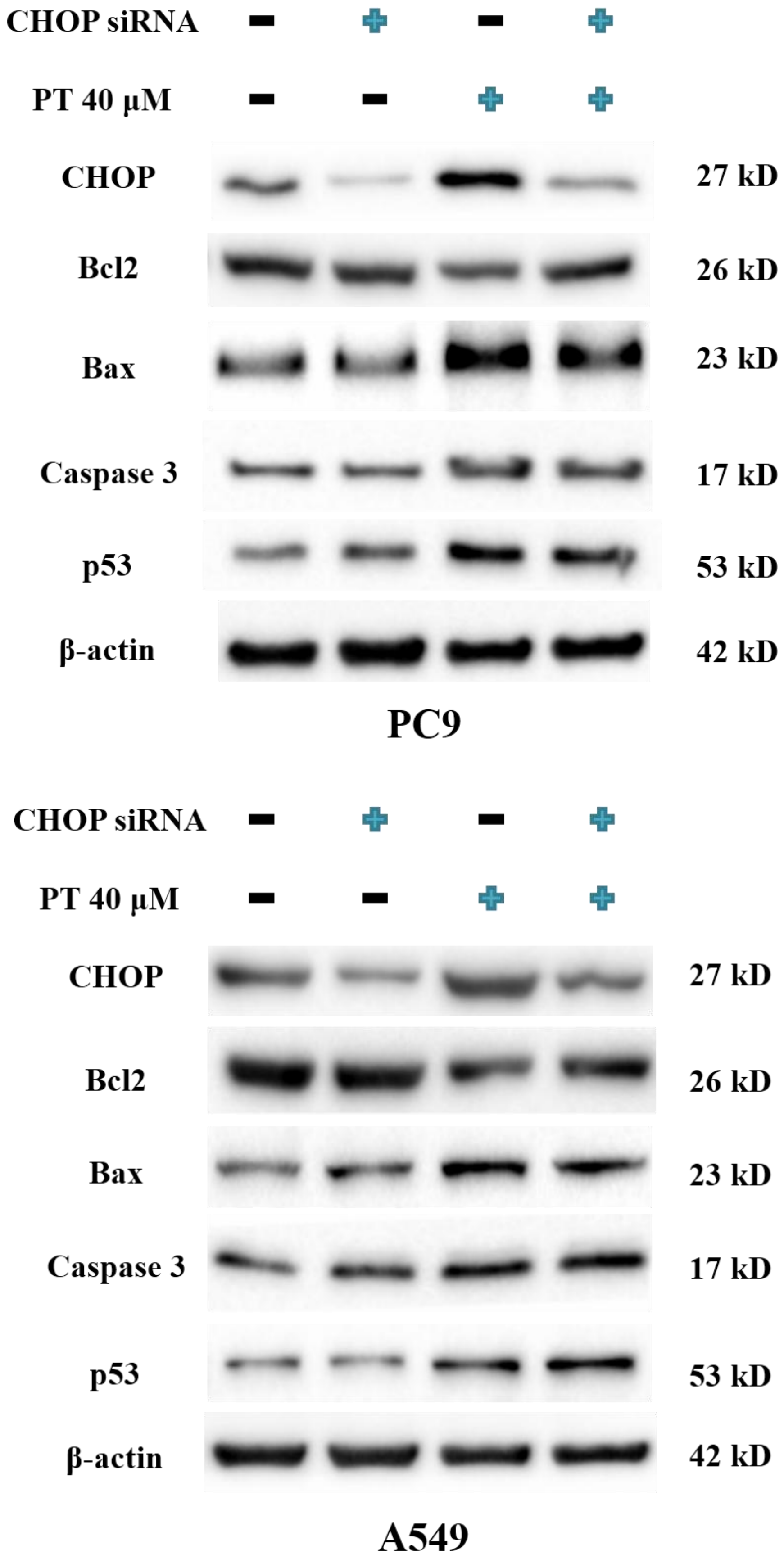

Supplementary Figure 7: The full length blots in Fig. 7D

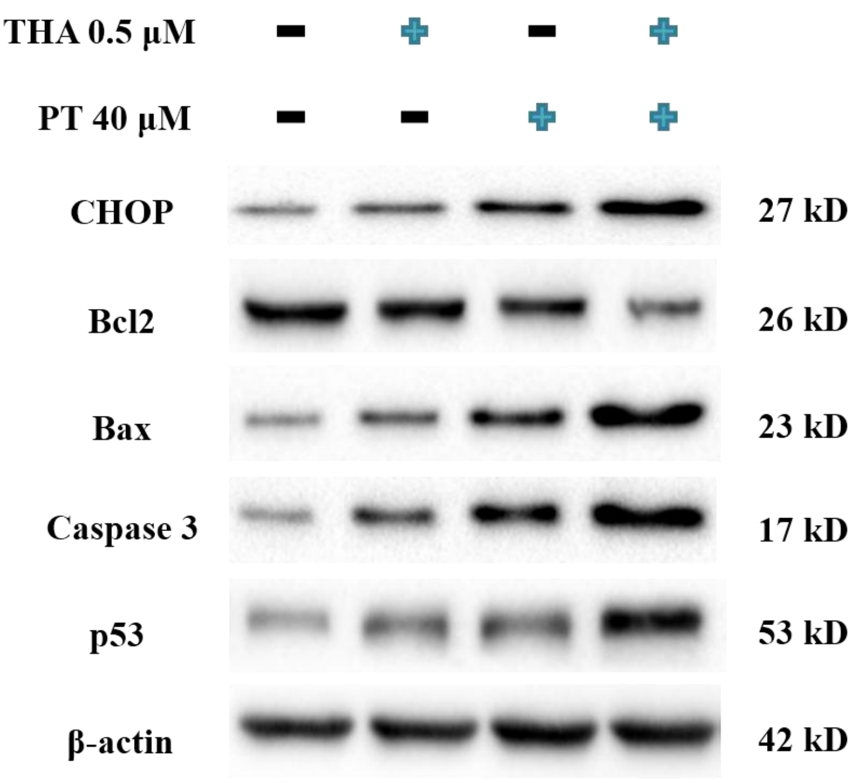

PC9

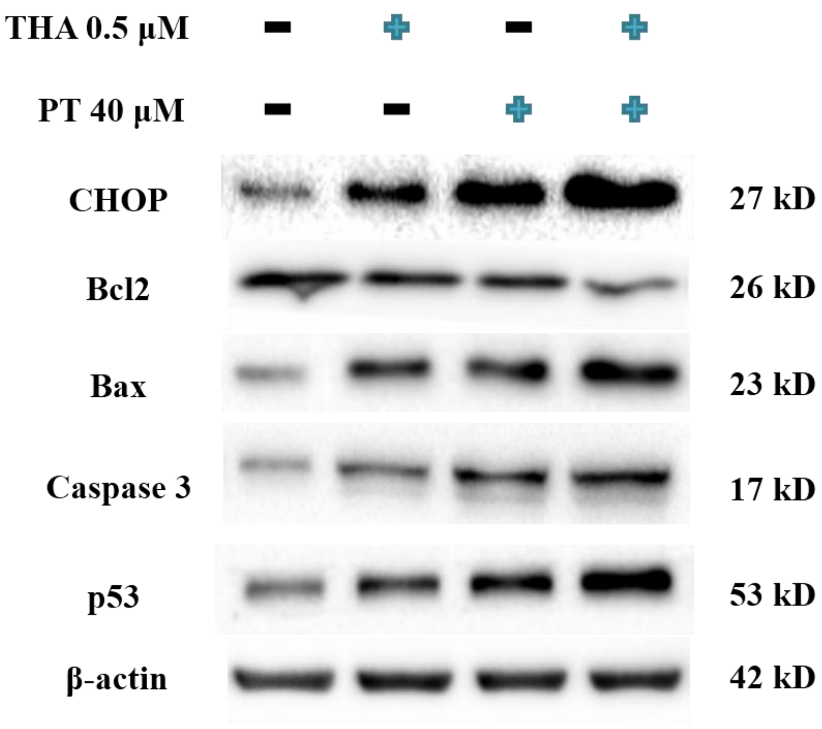

A549

Supplementary Figure 8: The full length blots in Fig. 8B

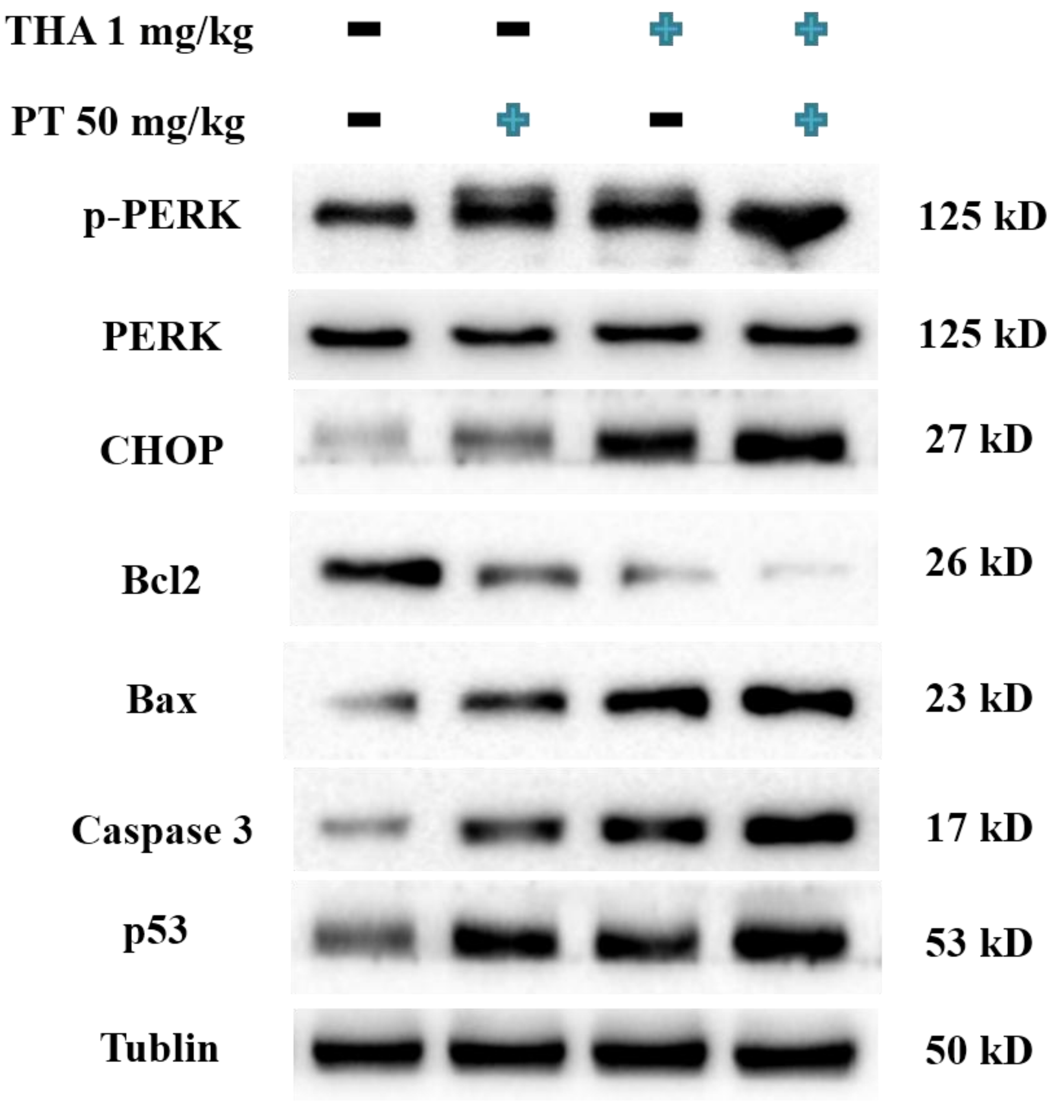

Supplement: Supplementary file 1 — Supplementary Information [file 41598_2017_8547_MOESM1_ESM.pdf]
